# Sugarcane mosaic virus mediated changes in cytosine methylation pattern and differentially transcribed fragments in resistance-contrasting sugarcane genotypes

**DOI:** 10.1371/journal.pone.0241493

**Published:** 2020-11-09

**Authors:** Marcel Fernando da Silva, Marcos Cesar Gonçalves, Michael dos Santos Brito, Cibele Nataliane Medeiros, Ricardo Harakava, Marcos Guimarães de Andrade Landell, Luciana Rossini Pinto

**Affiliations:** 1 Biologia Aplicada à Agropecuária, Faculdade de Ciências Agrárias e Veterinárias (FCAV) Universidade Estadual Paulista “Júlio de Mesquita Filho”, Jaboticabal, São Paulo, Brazil; 2 Crop Protection Research Centre, Instituto Biológico, São Paulo, Brazil; 3 Departamento de Ciência e Tecnologia, Instituto de Ciência e Tecnologia da Universidade Federal de São Paulo, São José dos Campos, São Paulo, Brazil; 4 Centro de Cana, Instituto Agronômico, Ribeirão Preto, São Paulo, Brazil; ICAR - National Research Center on Plant Biotechnology, INDIA

## Abstract

*Sugarcane mosaic virus* (SCMV) is the causal agent of sugarcane mosaic disease (SMD) in Brazil; it is mainly controlled by using resistant cultivars. Studies on the changes in sugarcane transcriptome provided the first insights about the molecular basis underlying the genetic resistance to SMD; nonetheless, epigenetic modifications such as cytosine methylation is also informative, considering its roles in gene expression regulation. In our previous study, differentially transcribed fragments (DTFs) were obtained using cDNA-amplified fragment length polymorphism by comparing mock- and SCMV-inoculated plants from two sugarcane cultivars with contrasting responses to SMD. In this study, the identification of unexplored DTFs was continued while the same leaf samples were used to evaluate SCMV-mediated changes in the cytosine methylation pattern by using methylation-sensitive amplification polymorphism. This analysis revealed minor changes in cytosine methylation in response to SCMV infection, but distinct changes between the cultivars with contrasting responses to SMD, with higher hypomethylation events 24 and 72 h post-inoculation in the resistant cultivar. The differentially methylated fragments (DMFs) aligned with transcripts, putative promoters, and genomic regions, with a preponderant distribution within CpG islands. The transcripts found were associated with plant immunity and other stress responses, epigenetic changes, and transposable elements. The DTFs aligned with transcripts assigned to stress responses, epigenetic changes, photosynthesis, lipid transport, and oxidoreductases, in which the transcriptional start site is located in proximity with CpG islands and tandem repeats. Real-time quantitative polymerase chain reaction results revealed significant upregulation in the resistant cultivar of aspartyl protease and VQ protein, respectively, selected from DMF and DTF alignments, suggesting their roles in genetic resistance to SMD and supporting the influence of cytosine methylation in gene expression. Thus, we identified new candidate genes for further validation and showed that the changes in cytosine methylation may regulate important mechanisms underlying the genetic resistance to SMD.

## Introduction

Sugarcane (*Saccharum* spp.) is the raw material used for sugar and energy production in the tropics [1, 2]. Modern commercial sugarcane cultivars are interspecific hybrids with 70–80% of the genome derived from the noble cane *Saccharum officinarum* (2n = 80, x = 10)—having high sucrose content—10–20% from *Saccharum spontaneum* (2n = 40–128, x = 8)—which confers environmental adaptability, disease resistance, and ratooning capacity—and 10% from recombinants [3, 4]. Globally, sugarcane production is affected by diverse biotic and abiotic stresses [5], with sugarcane mosaic disease (SMD) being one of the main diseases affecting this crop. SMD has been widely reported in the major sugarcane-growing countries and is caused by viruses of the family *Potyviridae*, namely, sugarcane mosaic virus (SCMV) and sorghum mosaic virus of the genus *Potyvirus* and sugarcane streak mosaic virus of the genus *Poacevirus* [6]. In Brazil, only SCMV has yet been reported in sugarcane [7, 8]. The control of SMD relies mainly on breeding for genetic resistance, which highlights the importance of understanding its molecular basis for sugarcane breeding programs [6, 7].

Sugarcane breeding still relies mostly on conventional methods owing to its very complex and polyploid genome, which imposes challenges to sugarcane genetics knowledge [3, 9]. Sequence data have become increasingly available in the last few years, comprising modern sugarcane cultivars, e.g., expressed sequence tags (ESTs) [10]; long-read libraries [11]; the recently released gene space assembly [12] for SP80-3280; the mosaic monoploid reference for R570 [3]; and the *S*. *spontaneum* AP85-441 haploid assembly [9]. Differentially expressed gene profiling has been used for the identification of genes and pathways related to sugarcane biological features [13, 14]; nonetheless, few studies have investigated the changes in the transcriptome of sugarcane infected by mosaic-causing viruses [15, 16]. Among the available techniques for transcriptome investigation, cDNA-amplified fragment length polymorphism (cDNA-AFLP) has been applied for candidate gene identification by performing BLASTN alignments with differentially transcribed fragments (DTFs) under various contrasting conditions [15, 17].

Important roles in transcriptional regulation in eukaryotes have been reported for epigenetic processes such as DNA methylation, RNA interference (RNAi), and histone modifications [18–20]. In addition, growing evidence suggested a correlation between changes in DNA methylation patterns and plant defense gene expression [21, 22]. In plants, DNA methylation occurs on three sequence contexts—CpG, CpNpG, and CpNpN—where N stands for A, C, or T and involves the addition of a methyl group from *S*-adenosylmethionine to the 5′ position of cytosine, leading to the conversion of cytosine to 5-methylcytosine (5mC) [23]. The methods for DNA methylation investigation are classified according to three detection principles: endonuclease digestion, affinity enrichment, and bisulfite conversion [24].

Methylation-sensitive amplification polymorphism (MSAP) is an endonuclease-based technique that allows the assessment of cytosine methylation state in numerous random loci over the genome, allowing its use in non-model organisms [24, 25]. The technique is a modification of AFLP [26] and is based on parallel digestions with HpaII and MspI isoschizomers, with differential susceptibility to cytosine methylation at the CCGG motif, used as frequent cutter enzyme, in combination with an endonuclease indifferent to cytosine methylation as the rare cutter, e.g., EcoRI [25, 27].

According to Schultz et al. [27], HpaII cleaves both unmethylated and hemimethylated CCGG motifs at the external cytosine (5mCCGG), whereas MspI cleaves unmethylated as well as hemi- or fully methylated CCGG motifs at the internal cytosine (C5mCGG). MSAP was successfully used to investigate the changes in cytosine methylation patterns under abiotic and biotic stresses [28, 29] as well as determine the association between differentially methylated fragments (DMFs) and their function by using cloning, sequencing, and aligning in databases by using the BLAST tool [28, 30].

Taking the above into account, the present study aimed to (1) investigate the cytosine methylation patterns at the CCGG motifs under SCMV infection by using MSAP and its effects on gene expression of the transcripts associated with DMFs by using reverse transcriptase real time-quantitative polymerase chain reaction (RT-qPCR); (2) expand the identification of new DTFs and validate the results by using RT-qPCR. The MSAP analysis results and DMF alignments suggested a biological relevance of cytosine methylation in the interaction between sugarcane and SCMV, whereas RT-qPCR revealed potential roles of this epigenetic mark in the regulation of gene expression. Relevant functions of DTFs, including potential interplays with epigenetic pathways, were also identified. The validation of three DTFs by using RT-qPCR revealed an overall disagreement with previous cDNA-AFLP findings, with significant upregulation of the DTF assigned to VQ proteins.

## Material and methods

### Plant material and experimental design

Leaf samples used for DNA extraction were derived from a previous experiment performed under greenhouse conditions by Medeiros et al. [15]. The experimental design was a completely randomized factorial 2 x 2 x 3 as follows: (a) two sugarcane cultivars from the IAC Sugarcane Breeding Program, i.e., IACSP95-5000 resistant and IAC91-1099 susceptible to SMD; (b) two treatments, i.e., SCMV mechanical inoculation (s.i) and mock inoculation (m.i); and (c) three time points, i.e., 24, 48, and 72 h post-inoculation (hpi). For each cultivar, 36 plantlets obtained by meristem tip culture were indexed as virus-free by using RT-PCR and specific primers for the SCMV capsid protein [31]. At 1-month-old, half of the 36 sugarcane plantlets were mechanically inoculated (s.i treatment), and the remaining were mock-inoculated (m.i treatment). Leaves of *Sorghum bicolor* (L.) ‘Rio’ plantlets, previously inoculated with SCMV-Rib1, an aggressive SCMV strain described by Gonçalves et al. [32], and showing typical mosaic symptoms, served as the virus inoculum. The virus inoculum was prepared by grinding the sorghum leaves in 0.01 M phosphate buffer, pH 7.2 at 4°C, at a 1:10 (mg:mL) ratio, and then mixed with abrasive silicon carbide (600 mesh) for the mechanical inoculation (s.i treatment) of the first leaf with visible dewlap from the top to bottom of the stalk, i.e., the +1 leaf. Conversely, for the m.i treatment, mock inoculation with phosphate buffer plus silicon carbide was used. The same three biological replicates of each cultivar, treatment, and time points were used either for RNA or DNA extraction.

### DNA extraction

Twelve pools (IAC91-1099 m.i 24 hpi, IAC91-1099 s.i 24 hpi, IAC91-1099 m.i 48 hpi, IAC91-1099 s.i 48 hpi, IAC91-1099 m.i 72 hpi, IAC91-1099 s.i 72 hpi, IACSP95-5000 m.i 24 hpi, IACSP95-5000 s.i 24 hpi, IACSP95-5000 m.i 48 hpi, IACSP95-5000 s.i 48 hpi, IACSP95-5000 m.i 72 hpi, and IACSP95-5000 s.i 72 hpi) each consisting of 150 mg of the respective +1 leaf from three biological replicates were used for total genomic DNA extraction by using the DNAeasy plant extraction kit (Sigma). DNA was quantified by comparison with DNA λ fage in 0.8% 1X TBE agarose gel stained with ethidium bromide (1 μg mL^-1^).

### MSAP derived markers

MSAP markers were obtained according to Lei et al. [33] and adapted for sugarcane, as described by Francischini et al. [34]. In brief, each DNA pool was digested separately with 2.5 U of EcoRI–HpaII or EcoRI–MspI restriction enzyme combination at 37°C for 4 h, and adaptor ligation was performed at 37°C for 16 h. The reaction was diluted 6× in Mili-Q water for the pre-selective amplification reaction, and then 5 μL aliquot of this reaction was diluted 10x in Mili-Q water for selective amplification by using HpaII–MspI/EcoRI primers with three selective nucleotides at the 3′-terminal and fluorescently labeled with IRD 700 or IRD 800 dyes. Aliquots of PCR products from HpaII–MspI/EcoRI (700) and HpaII–MspI/EcoRI (800) selective primer combinations were mixed, added to loading buffer (LiCor, Bioscience), diluted five times in Mili-Q water, denatured for 5 s at 95°C, and separated on 6% denaturing polyacrylamide gel by using a DNA analyzer (Infrared 4300 DNA Analyzer; LiCor Bioscience). In all, 29 selective primer combinations were evaluated (S1 Table, S1 Fig).

### MSAP data analysis

The MSAP markers were genotyped for presence (+) and absence (-) by comparing side-by-side m.i and s.i treatments (m.i EcoRI–HpaII, m.i EcoRI–MspI, s.i EcoRI–HpaII, and s.i EcoRI–MspI) at the three time points. The classification of the methylation status of restriction sites (5′-CCGG-3′) was based on Schultz et al. [27], wherein (++/++), (+-/+-), (-+/-+), and (—/—) were interpreted as unmethylated, hemimethylated at the external cytosine, fully or hemimethylated at the internal cytosine, and either fully methylated at the external cytosine or methylated in both cytosines or absence of restriction sites, respectively. The proportion of MSAP loci methylation was determined according to Abid et al. [28]. Band pattern differences between m.i and s.i samples within the same cultivar and time points were considered as DMFs.

MSAP band presence and absence were also evaluated using MSAP R package [35] in R 3.0.3 program (R Core Team, 2014). For this analysis, MSAP data were distributed in four groups, i.e., IAC91-1099 m.i, IAC91-1099 s.i, IACSP95-5000 m.i, and IACSP95-5000 s.i, each encompassing the time points of 24, 48, and 72 hpi. The MSAP bands were classified as either methylation-susceptible loci (MSL) or nonmethylated loci (NML) based on methylation frequencies, determined on the basis of EcoRI–HpaII(+)/EcoRI–MspI(-) or EcoRI–HpaII(-)/EcoRI–MspI(+) presence above or below a 5% default error threshold, respectively [35]. The MSL and NML information content was determined using Shannon’s diversity index, and differences were tested using the Wilcoxon rank-sum test at 5% probability. By using the resulting matrices of MSL and NML loci, we assessed the relative distance among groups (treatments) by using principal coordinate analysis (PCoA) via METABOANALYST v.4.0 [36] software.

### Isolation of DMFs and DTFs

Four selective combinations showing DMFs were run on 6% denaturing polyacrylamide gel and silver-stained according to Creste et al. [37]. Randomly selected DMFs were excised from polyacrylamide gel and eluted in 50 μL of TE buffer (10 mM Tris-HCl, 1.0 mM EDTA, pH 8.0), incubated at 60°C for 2 h, and centrifuged for 10 s for polyacrylamide separation. The DMFs were reamplified from 10 μL of the elutant by using the same primers and PCR cycling conditions of the respective selective amplification. Previously isolated DTFs were reamplified using the same method described for DMFs. The reamplified PCR products were separated on 1% 1X TBE agarose gel and further eluted and purified using Wizard^®^ SV Gel and PCR Clean-up system “kit” (Promega, USA).

### Cloning and sequencing

The purified PCR products were cloned into the pGEM-T easy vector (Promega, USA) and transformed into *Escherichia coli* DH10B competent cells, according to manufacturer’s instructions, and individual clones were sequenced at the Center for Biological Resources and Genomic Biology by using the Sanger method.

### Sequence analysis

BLASTN homology search was performed for DMFs and DTFs against the following databases: the mosaic monoploid genome of R570 available at CIRAD database (http://sugarcane-genome.cirad.fr/content/blast); the current sequence information on SP80-3280, namely, the 373k gene space assembly available at NCBI (https://www.ncbi.nlm.nih.gov/) under the accession number ASM869266v1; the RNA-seq transcripts and ESTs available at SUCEST-FUN (http://sucest-fun.org/wsapp/); the draft genome available at CTBE (http://bce.bioetanol.cnpem.br/ctbeblast/); and the *S*. *spontaneum* AP85-441 haploid assembly obtained by downloading the online database cited in Zhang et al. [9] and by using the free-to-use public server hosted by the Galaxy project (https://usegalaxy.org/). We favored alignments with higher identity (ident), query cover, and the lowest e-values among these databases. When similar results were observed for these parameters, we preferred that the alignments were obtained using the CIRAD database because of its better description of transcripts.

The functional categories of these transcripts were searched in UniProt database (http://www.uniprot.org/) by using BLASTX, by prioritizing Gene Ontology (GO) terms from the “biological process” category, followed by protein families (pfam) and GO terms from the “molecular function” category. An e-value cut off of 1e-5 was adopted for both BLASTN and BLASTX tools. The transcriptional start site (TSS) was found via BLASTX alignments between transcripts and proteins from their respective sugarcane databases, allowing frame alignment corresponding to the start of the protein. These transcripts were translated using the online ExPASy translation tool (https://web.expasy.org/translate/) for retrieving the nucleotide sequence encompassing the start codon from the corresponding open reading frame. Based on these alignments, we also inferred whether DMFs and DTFs aligned to regions corresponding to exons, i.e., 5′-untranslated region (UTR), coding sequence (CDS), and 3′-UTR, or to introns, except for sequences from the CIRAD database, in which these regions are already discriminated. The transcripts homologous to DMFs and DTFs were further aligned to sugarcane genomic sequences from their respective databases. In the case of SUCEST ESTs, the genomic sequences were aligned against either the CTBE draft genome or the 373k gene space assembly, whichever showed higher identity, query cover, and the lowest e-values. We retrieved a 3 kb region upstream of the TSS from these alignments and scanned it for putative transcription factor–binding sites, CpG islands, and tandem repeats by using the PlantPAN 3.0 database (http://plantpan.itps.ncku.edu.tw/promoter.php), all of which were positioned in relation to the TSS. Considering that the DMFs could be matched with genomic regions corresponding to the transcription factor–binding sites, adjacent transcripts were investigated in two adjacent locations up to 3000 bp, with the selection of those in which the 5′-UTR was downstream the putative DMF alignments within this range. Subsequently, these transcripts were analyzed using Uniprot and scanned for putative cis-regulatory sequences, CpG islands, and tandem repeats, as described above for the transcripts homologous to DMFs and DTFs.

### cDNA synthesis and qPCR analysis

According to our previous cDNA-AFLP results [15], a higher number of DTFs were observed at 24 and 72 hpi. Therefore, we performed RT-qPCR validation in the following experimental factor combinations: IAC91-1099 24 hpi (m.i), IAC91-1099 24 hpi (s.i), IAC91-1099 72 hpi (m.i), IAC91-1099 72 hpi (s.i), IACSP95-5000 24 hpi (m.i), IACSP95-5000 24 hpi (s.i), IACSP95-5000 72 hpi (m.i), and IACSP95-5000 72 hpi (s.i). Total RNA was previously isolated from the +1 leaf of each biological replicate by Medeiros et al. [15], and 1μg was treated with RNase-free DNase (Promega, Fitchburg WI, USA) and reverse transcribed using the GoScript Reverse Transcription System (Promega) kit with an oligo (dT)_20_ primer, according to manufacturers’ protocols. Based on the alignments of DMFs and DTFs with matches representing potential roles in SMD resistance pathways, we performed quantitative expression analysis by using RT-qPCR for the corresponding genes. Primers were designed using PrimerQuest (https://eu.idtdna.com/primerquest/home/index) and Primer-BLAST tools (http://www.ncbi.nlm.nih.gov/tools/primer-blast), whereas primer quality was estimated using Netprimer software (http://www.premierbiosoft.com/NetPrimer/AnalyzePrimerServlet). The RT-qPCR consisted of 3 μL of (1:10) diluted cDNA, 5 μL of SYBR Green Power Master Mix (Applied Biosystems), and 0.2 μM of each forward and reverse primers in a total reaction volume of 10 μL conducted on an Applied Biosystems StepOnePlus System (Foster City CA, USA) under the following thermal cycling conditions: initial denaturation at 95°C for 20 s, followed by 40 cycles at 95°C for 3 s, and 60°C for 30 s. The product specificity and reaction efficiency were, respectively, determined using melting curve analysis and LinReg PCR program [38] after RT-qPCRs by using four bulks of cDNA diluted 1:10, each comprising three biological replicates and two time points from the validation experiment. The selected primers were validated using the three aforementioned biological and technical replicates, yielding a total of 72 RT-qPCRs plus three non-template controls (NTCs) per gene. Two reference genes, uridylate kinase (UK) and ubiquitin-conjugating enzyme 18 (UBC18) [39], were used for normalization. The relative level of expression was calculated according to Taylor et al. [40] and tested using unpaired (homoscedastic) Student’s *t*-test with two‐tailed distribution.

## Results and discussion

### Alterations in DNA methylation pattern

In all, 1,131 MSAP loci, varying from 11 to 59 per selective primer combination, were observed (S1 Appendix). The frequency of the four types of DNA methylation patterns is shown in Table 1.

**Table 1:** MSAP pattern frequency for cultivars IAC91-1099 and IACSP95-5000 under mock inoculation (m.i) and mechanical inoculation with SCMV (s.i) treatments at 24, 48, and 72 hpi. (+) and (−) represent presence and absence of bands, respectively.

MSAP pattern	IAC91-1099 (24 hpi)		IAC91-1099 (48 hpi)		IAC91-1099 (72 hpi)	
*Hpa*II/*Msp*I	m.i	s.i	m.i	s.i	m.i	s.i
++	748	758	757	751	733	731
+-	72	61	55	86	62	66
-+	207	202	227	197	213	218
--	104	110	92	97	123	116
Total	1131	1131	1131	1131	1131	1131
Full methylation (%)^a^	27.5	27.6	28.2	26.0	29.7	29.5
Hemi-methylation (%)^b^	6.4	5.4	4.9	7.6	5.5	5.8
Total of methylated bands (%)^c^	33.9	33.0	33.1	33.6	35.2	35.4
MSAP pattern	IACSP95-5000 (24 hpi)		IACSP95-5000 (48 hpi)		IACSP95-5000 (72 hpi)	
*Hpa*II/*Msp*I	m.i	s.i	m.i	s.i	m.i	s.i
++	709	745	758	741	727	765
+-	49	54	48	65	70	47
-+	220	192	186	189	190	190
--	153	140	139	136	144	129
Total	1131	1131	1131	1131	1131	1131
Full methylation (%)^a^	33.0	29.4	28.7	28.7	29.5	28.2
Hemi-methylation (%)^b^	4.3	4.8	4.2	5.7	6.2	4.2
Total of methylated bands (%)^c^	37.3	34.1	33.0	34.5	35.7	32.4

^a^: {[Loci with inner cytosine methylation (-+) + Loci with full methylation at both cythosines (—)]/Total of Loci}*100; ^b^: {[Loci hemi-methylated at the external cytosine (+-)]/Total of Loci} *100

^c^: Full methylation (%)^a^ + Hemi-methylation (%)^b^.

Total methylation of CCGG sequences ranged from 33.1% to 35.2% in IAC91-1099 and from 33.0% to 37.3% in IACSP95-5000, under m.i treatment, and from 33% to 35.4% in IAC91-1099 and from 32.4% to 34.5% in IACSP95-5000, under s.i treatment. The total methylation of CCGG sequences varied notably among the time points, especially for IACSP95-5000. The highest alterations in total cytosine methylation due to SCMV infection for IACSP95-5000 were reduction of 3.2% (24 hpi), increase of 1.5% (48 hpi), and decrease of 3.3% (72 hpi). Conversely, changes in cytosine methylation in the susceptible cultivar IAC91-1099 occurred mainly at 48 hpi, with a decrease of 2.2% in full methylation (which involved inner cytosine methylation of the CCGG motif) and an increase of 2.7% in external cytosine hemi-methylation, resulting in an increase of 0.5% of the total cytosine methylation. These results suggest that the inoculation of SCMV caused a switch in methylation from inner to outer cytosine.

The proportion of methylated loci was similar to that (35%) reported by Grativol et al. [41] in the genome of the sugarcane cultivar SP70-1143 by methyl filtration with McrBC endonuclease digestion, which assesses all three cytosine methylation contexts [42, 43]. The similarity in findings between our study and that of Grativol et al. [41] suggests either a preponderance of cytosine methylation at the CpG and CpNpG contexts in sugarcane or higher genome methylation levels of the cultivars investigated under our experimental conditions, since the CpNpN context was not assessed.

The relationship between the changes in cytosine methylation and SCMV infection over the three time points was based on the classification of the 1,131 MSAP loci into 24 classes (Table 2), i.e., eight classes for MSAP pattern with stable bands; four classes for DMFs responsive only to time points; six classes for hypomethylated DMFs, and six for hypermethylated DMFs in response to SCMV inoculation and time points.

**Table 2:** Frequency of MSAP patterns and changes under SCMV infection and time points for the cultivars IAC91-1099 and IACSP95-5000. (+) and (−) represent presence and absence of bands, respectively.

Response	(m.i)^a^		(s.i)^a^		Cultivars					
	*Hpa*II	*Msp*I	*Hpa*II	*Msp*I	IAC91-1099			IACSP95-5000		
No change^b^										
	+	+	+	+	665			662		
	+	-	+	-	24			12		
	-	+	-	+	139			131		
	-	-	-	-	59			98		
Subtotal					887			903		
					Time points					
					24 h	48 h	72 h	24 h	48 h	72 h
Time points^c^										
	+	+	+	+	11	9	9	4	12	10
	+	-	+	-	7	7	2	7	5	6
	-	+	-	+	6	10	11	11	10	9
	-	-	-	-	5	3	7	11	6	8
Subtotal					29	29	29	33	33	33
SCMV inoculation^d^										
Hypomethylation	+	-	+	+	6	4	12	11	10	32
	-	+	+	+	20	34	10	42	7	17
	-	-	+	+	2	0	1	1	0	8
	-	-	+	-	10	6	14	20	9	5
	-	-	-	+	6	9	21	5	5	15
Change^e^	-	+	+	-	1	2	11	3	4	3
Subtotal					45	55	69	82	35	80
Hypermethylation	+	+	+	-	4	34	9	4	23	7
	+	+	-	+	13	6	13	13	11	11
	+	+	-	-	1	4	3	1	0	1
	+	-	-	-	19	5	6	9	5	3
	-	+	-	-	4	11	20	3	6	9
Change^e^	+	-	-	+	1	2	12	2	4	3
Subtotal					42	62	63	32	49	34
No change^f^	+	+	+	+	54	39	34	25	50	36
	+	-	+	-	15	13	6	8	12	14
	-	+	-	+	37	31	22	30	28	21
	-	-	-	-	22	15	21	18	21	10
Subtotal					128	98	83	81	111	81
Total					1131	1131	1131	1131	1131	1131

^a^: Mock inoculated (m.i), SCMV inoculated (s.i)

^b^: Loci nonresponsive neither to time point nor to SCMV inoculation

^c^: Loci exclusively responsive to time point

^d^: Loci responsive to SCMV via hypermethylation, hypomethylation

^e^: Loci with changes in methylation between inner and outer cytosine

^f^: Loci nonresponsive neither to time points nor to SCMV inoculation in at least one time point.

Changes in cytosine methylation in response to SCMV were found to differ between the susceptible and resistant cultivars. The total hypomethylation of cultivar IAC91-1099 increased from 24 to 48 hpi and from 48 to 72 hpi, mostly because of the DMF patterns (-+/++) and (—/-+), respectively. Cultivar IACSP95-5000 showed decreased hypomethylation from 24 to 48 hpi, because of DMF patterns (-+/++) and (—/+-), and an increase from 48 to 72 hpi because of the DMF pattern (+-/++). This cultivar also showed increased hypermethylation from 24 to 48 hpi and decreased hypermethylation from 48 to 72 hpi because of the changes in the frequencies of the DMF pattern (++/+-). Conversely, in cultivar IAC91-1099, hypermethylation increased from 24 to 48 hpi, resulting from the decrease in DMF patterns (++/-+) and (+-/—) and increase in DMF patterns (++/+-) and (-+/—). The DMF patterns (-+/+-) and (+-/-+), which represent the switches in the methylated cytosine position in the CCGG motif, had relatively low frequency in IAC91-1099 at 48 hpi; therefore, they did not cause the switch in methylation from the inner to outer cytosine (Table 1). This change in the position was caused by the decrease of the hypomethylated DMF pattern (-+/++), which involves internal cytosine methylation, and the increase in hypermethylated DMF pattern (++/+-), involving external cytosine methylation (Table 2).

### MSAP global analysis

**Fig 1:**
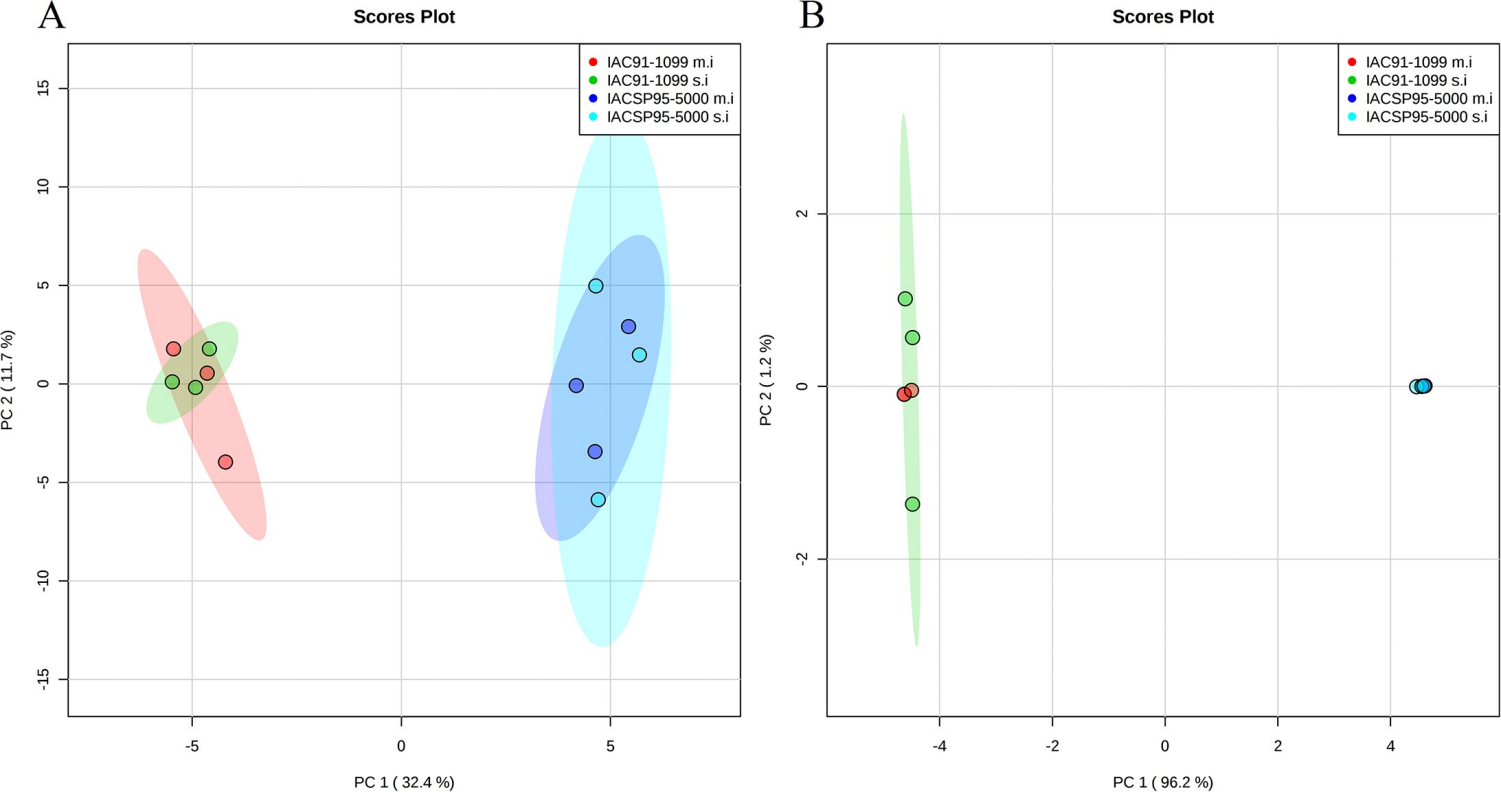
Principal coordinate analysis (PCoA) for A: methylation-susceptible loci (MSL) and B: nonmethylated loci (NML), representing epigenetic and genetic differences among groups, respectively.

The extent of cytosine methylation across all MSAP loci was assessed using the R MSAP package, revealing a total of 457 MSL and 674 NML, which corresponded to a proportion of 40%. The polymorphism proportion for MSL and NML was 59% (272 loci) and 13% (90 loci), respectively, representing the differences among and within groups. The Shannon diversity index (I) values for MSL (I = 0.58 ± 0.12) and NML (I = 0.67 ± 0.088) with 1% significance by the Wilcoxon sum rank test (W = 3756; *P*< 0.0001) revealed higher genetic variations among groups in relation to epigenetic variations. According to the PCoA analysis, each cultivar formed distinct groups either for MSL or NML, but with overlap of m.i and s.i groups. Intragroup variation was pronounced in MSL for both the cultivars, indicating higher diversity among the time points for these loci. The first two principal coordinates accounted for 50.3% of the total variation for MSL, whereas they explained 97.4% of the variation for NML (Fig 1).

The high frequency of MSAP bands not responsive to SCMV infection (Table 2) and the overlap between m.i and s.i groups from PCoA analysis for MSL (Fig 1) suggested that only mild changes in cytosine methylation occurred in sugarcane challenged with SCMV inoculation. Plant species have robust mechanisms for the maintenance of DNA methylation, such as retargeting and RNAi mechanisms; the failure of these mechanisms is a major cause for the changes in DNA methylation [44]. Changes in cytosine methylation are also associated with stress responses [45], possibly with implications on plant immunity [23, 46].

The higher frequency of hypomethylated DMFs in cultivar IACSP95-5000 at 24 and 72 hpi, respectively, (Table 2) is indicative of stress response. The relevance of these alterations is revealed by studies on challenges by pathogens, which cause the hypomethylation of the leucine-rich repeat (LRR) loci [47], associated with the effector-triggered immunity (ETI) pathways—one of the major branches in plant innate immunity—and of regions flanking both the ends of defense-related genes, enhancing their expression [46].

### Sequencing of DMFs

In all, 19 DMFs were excised from acrylamide gels, cloned, and sequenced. The sequences ranged from 45 to 238 bp after adapter trimming. The BLAST results revealed 17 significant alignments in at least one sugarcane database (Table 3, S1 File).

**Table 3:** BLASTN analysis of 19 differentially methylated fragments (DMFs) and BLASTX analysis of the sugarcane transcripts. (+) and (−) represent presence and absence of bands, respectively.

DMF (Time points/Selective combination)	Size (bp)	MSAP pattern	BLASTN (Ident/Query cover/ e-value/Position) ^a^	Uniprot (Species/Annotation score/Ident/Query cover/e-value)^e^	Annotation (Accession)
Hypomethylation					
1099_01 (24 h/*Eco*RIACA/*Msp*ITTG)	80	+-/++	SCSP803280_000073600 (97.56/100.00/4e-38/Genomic)^b^		
1099_02 (24 h/*Eco*RIACA/*Msp*ITTG)	82	+-/++	SCSP803280_000073600 (97.56/100.00/3e-32/Genomic)^b^		
1099_03 (24 h *Eco*RIACA/*Msp*ITTG)	45	+-/++	Sh_018M23_contig-1_g000070 (97.73/97.00/7e-20/5'-UTR)^e^	Q64M78 (*Oryza sativa*/2/76.34/43.00/4e-128)	mRNA splicing, via spliceosome (GO:0000398)^f^
1099_04 (24 h *Eco*RIACA/*Msp*ITTG)	117	--/+-	SCSP803280_000016069 (94.02/100.00/3e-49/Genomic)^b^		
5000_05 (24 h *Eco*RIACA/*Msp*ITAG)	86	--/++	Sspon.08G0008750-3D (97.65/98.00/4e-39/CDS and Intron)^d^	A0A1E5UIV7 (*Dichanthelium oligosanthes*/1/73.13/5.00/1.1e-22)	Myb-like DNA-binding domain (PF00249)^g^
5000_06 (24 h *Eco*RIACA/*Msp*ITAG)	114	--/-+	Sh_206E04_g000020 (86.17/81.00/1e-22/Intron)^e^	C5XWZ0 (*Sorghum bicolor*/1/97.27/22.00/3e-167)	protein phosphorylation (GO:0006468)^f^
5000_07 (24 h *Eco*RIACA/*Msp*ITTG)	101	--/++	SP803280_c96114_g1_i1 (93.81/94.00/1e-42/Exon)^d^	A0A1D6F4S8 (*Zea mays*/1/40.54/27.00/9e-06)	Zinc finger, C3HC4 type (PF00097)^g^
5000_08 (48 h *Eco*RIACA/*Msp*IACA)	119	--/-+	Sh_143N13_contig-1_g000100 (100.00/14.00/0.018)^c^		
5000_09 (48 h *Eco*RIACA/*Msp*IACC)	125	+-/++	Sspon.06G0001250-2C (71.29/80.00/2e-10/Intron)^d^	C5YJ64 (*Sorghum bicolor*/1/92.55/17.00/0.0)	cell surface receptor signaling pathway (GO:0007166)^f^
5000_10 (72 h *Eco*RIACA/*Msp*IACA)	183	--/-+	Sspon.02G0041100-1B (86.79/57.00/3e-34/Intron)^d^	A0A1J6KID8 (*Nicotiana attenuata*/1/22.22/8.00/3.80)	
1099_11 (48 h *Eco*RIACA/*Msp*ITAG)	160	--/-+	Sspon.05G0013670-1P (100.00/100.00/7e-82/CDS)^d^	A0A1B6PJT8 (*Sorghum bicolor*/1/85.54/52.00/0.0)	DNA binding (GO:0003677)^h^
Hypermethylation					
1099_12 (48 h *Eco*RIACA/*Msp*IACC)	222	-+/—	SCSP803280_000032883 (100.00/100.00/3e-115/Genomic)^b^		
5000_13 (24 h *Eco*RIACA/*Msp*IACA)	144	++/+-	Sh_241P15_contig-1_g000060 (97.22/100.00/2e-68/Intron)^c^	C5Z840 (*Sorghum bicolor*/1/96.76/74.00/0.0)	oligopeptide transmembrane transporter activity (GO:0035673)^h^
5000_14 (24 h *Eco*RIACA/*Msp*ITAG)	154	-+/—	SP803280_c104096_g2_i1 (96.58/94.00/2e-68/3'-UTR)^b^	C5Z2V8 (*Sorghum bicolor*/2/90.97/85.00/0.0)	Proteolysis (GO:0006508)^f^
5000_15 (24 h *Eco*RIACA/*Msp*ITAG)	104	++/+-	Sh_217F15_contig-1_g000090 (85.00/31.00/0.052)^c^		
5000_16 (48 h *Eco*RIACA/*Msp*ITAG)	238	-+/—	SP803280_c132337_g1_i1 (76.62/83.00/2e-39/CDS)^b^	B8BE31 (*Oryza sativa*/1/31.68/99.00/9e-26)	Reverse transcriptase; Reverse transcriptase-like (PF00078 /PF13456)^g^
5000_17 (72 h *Eco*RIACA/*Msp*ITTG)	82	-+/—	SCSP803280_000073600 (98.78/100.00/7e-38/Genomic)^b^		
5000_18 (72 h *Eco*RIACA/*Msp*IACC)	126	-+/—	SP803280_c89867_g1_i4 (93.16/92.00/1e-47/Intron)^b^	A0A1B6PTM2 (*Sorghum bicolor*/1/95.08/16.00/6e-38)	NB-ARC domain (PF00931)^g^
5000_19 (72 h *Eco*RIACA/*Msp*ITTG)	117	-+/—	SCSP803280_000016069 (98.29/100.00/3e-56/Genomic)^b^		

^a^: BLASTN alignment between DMFs and sugarcane sequences.

^b^: Hits with long-read libraries of SP80-3280 from CTBE database.

^c^: Hits with mosaic monoploid reference of R570 from CIRAD database.

^d^: Hits with *S*. *spontaneum* AP85-441 haploid assembly.

^e^: BLASTX alignment between sugarcane transcript and proteins from Uniprot database.

^f, g, h^: Gene Ontology (GO) terms from the "Biological Process" category, Pfam motifs, and GO terms from the "Molecular Function" category, respectively.

The GO terms “protein phosphorylation” and “cell surface receptor signaling pathway” assigned to the transcripts aligned with DMFs 5000_06 and 5000_09 suggested that the changes in cytosine methylation may play roles in plant immunity triggered by pathogen-associated molecular patterns (PAMPs)—the PAMP-triggered immunity (PTI)—since the detection of PAMPs involves surface-localized pattern recognition receptors (PRRs), whereas concurrent signal transmission occurs via phosphorylation cascades [48]. Moreover, the transcript aligned with DMF 5000_18 was assigned to the pfam motif NB-ARC, which is posited as a regulatory domain of nucleotide-binding leucine-rich repeat proteins [49], likely corresponding to antiviral ETI mechanisms [50]. Many proteins from the nucleotide-binding leucine-rich repeat class conferring resistance against viruses have been identified [50], whereas, only recently, the activation of plant PTI against viruses has been reported [51].

According to the GO annotation, the transcripts aligned to DMFs 5000_05, 1099_11, 5000_13, and 5000_14 are associated with plant responses to stress. For instance, DMFs 5000_05 and 1099_11 assigned to the pfam motif “Myb-like DNA-binding domain” also described as “SANT domain-containing protein”, which belongs to a transcription factor family involved in functions such as various types of biotic and abiotic stress responses, development, differentiation, metabolism, and defense [52, 53]. Furthermore, the GO term “proteolysis” assigned to the transcript SP803280_c104096_g2_i1, aligned with DMF 5000_14, suggests a role in autophagy pathways for cellular housekeeping in response to stresses by removing abnormal or misfolded proteins [54]. The transcript Sh_241P15_contig-1_g000060, aligned to DMF 5000_13, is described as an “NITRATE TRANSPORTER 1/PEPTIDE TRANSPORTER” (NRT1/PTR) protein and assigned to the GO term “oligopeptide transmembrane transporter activity.” NRT1/PTR proteins are known to be responsive to abiotic and biotic stresses [55], including infection by potyviruses [56].

Cytosine methylation is mosaically distributed across the genome of vegetal species, in association with transposable elements (TEs) and repetitive DNA in all sequence contexts, as well as with actively transcribed DNA regions and promoters, mostly at the CpG context [57, 58]. Intragenic cytosine methylation can be classified as intragenic heterochromatin, which is mostly located within TEs inserted into introns, and gene body methylation, primarily located within exons, but can also be found in introns [59]. Accordingly, our findings regarding DMF alignments, 1099_03, 5000_05, 5000_06, 5000_09, 5000_10, 1099_11, 5000_13, 5000_14, and 5000_16 may represent gene body methylation. Conversely, DMF 5000_18 aligned with SP803280_c89867_g1_i4 within a region that is not covered by the sorghum protein A0A1B6PTM2, assigned to the pfam motif NB-ARC, but is covered by a sugarcane protein from the CTBE database, deg7180000184898_g393, with pfam motifs "Retrotransposon gag protein" (PF03732), "Zinc knuckle" (PF00098), and "Reverse transcriptase" (PF00078), all of which are associated with retrotransposons [60], likely corresponding to intragenic heterochromatin methylation. TEs within introns are often repressed with epigenetic markers such as DNA methylation, as observed for the hypermethylated DMF 5000_18, and histone H3 Lysine 9 methylation (H3K9me) [59].

In addition to DMF 5000_18, a TE was also observed for 5000_16, owing to the assignment to the pfam motifs associated with TEs, i.e., "Reverse transcriptase" and "Reverse transcriptase-like" [60]. Environmental stresses may lead to the loss of DNA methylation in TEs, causing their spread throughout the genome, and new DNA methylation by RNA-directed DNA methylation processes result in DNA methylation pattern changes [19]. Promoter analysis for twelve DMFs by using PlantPAN revealed cis-regulatory sequences upstream the TSS (S2 Table).

PlantPAN analysis identified the cis-regulatory sequences AP2; ERF and EIN3; EIL for the DMFs 5000_06, 5000_09, and 5000_14. The first belongs to the APETALA2/ethylene-responsive factor (AP2/ERF) family, which is also responsible for development and stress responses [61]. ERF domains are reported to bind to GCC-box found in the promoters of many defense-related genes induced by ethylene [62]. In addition, ETHYLENE-INSENSITIVE 3 (EIN3)/EIN3-like (EIL) is a transcriptional factor responsible for ethylene-controlled transcriptional regulation of its immediate target genes, e.g., AP2/ERF [63, 64]. Ethylene has been extensively reported to be involved in plants’ response to biotic stresses, and, more recently, in the disruption of ethylene signaling by the potyviral protein NIa [65].

The cis-acting sequence bZIP, responsive to abiotic and biotic stresses [66], reinforces the roles of the transcripts associated with DMFs 1099_11 and 5000_13 in plant defense, which is in accordance with the findings of previous studies on SANT domain-containing protein [52, 53] and for proteins from the NRT1/PTR family [55, 56], in addition to suggesting the occurrence of retrotransposon regulation in response to stresses in the case of DMF 5000_16 [19]. It is also noteworthy the presence of the cis-acting sequence NAC; NAM upstream the transcript aligned with the DMF 5000_18 and assigned to ETI pathways. NAC (NAM/ATAF1/2/CUC2) represents a large family of plant-specific transcription factors, which play roles in different stages of plant immunity [67]. Lastly, a cis-acting sequence with putative functions of chromatin remodeling, i.e., zinc finger homeodomain (ZF-HD) [68], was observed upstream the transcript aligned with DMF 5000_05, suggesting regulations of stress responses by epigenetic pathways.

The alignment positions of DMFs 1099_03, 5000_06, 5000_10, 5000_13, 5000_14, and 5000_16 indicate cytosine methylation within the CpG islands. The transcripts aligned to these DMFs also harbored TSSs within the CpG islands, except for 1099_03 and 5000_10. For the latter two DMFs as well as for 1099_11 and 5000_18, the TSS was located in proximity to CpG islands, i.e., less than 1000 bp upstream. In addition, the proximity of tandem repeats to DMFs and to the respective TSS was observed for 5000_05 and 1099_11. The occurrence of cytosine methylation outside CpG islands in DMFs 1099_03, 5000_05, 1099_11, and 5000_18 is expected since it has been reported in plant species [69], whereas the proximity of CpG islands and tandem repeats to the TSS indicates methylation-dependent control of downstream transcripts [70].

The BLASTN search with the DMFs aligned to genomic regions against the mosaic monoploid genome of R570, draft genome of SP80-3280, and haploid assembly of *S*. *spontaneum* revealed that the DMFs 1099_01, 1099_04, 5000_17, and 5000_19 are upstream 5′-UTR transcripts in at least one database (S3 Table). Conversely, no 5′-UTR transcripts downstream DMF 1099_12 alignment were found within the 3000 bp range, and no 5′-UTR transcripts downstream of any of the DMFs aligned to genomic regions were observed when the sequences from the gene space assembly of SP80-3280 were used.

The alignment positions of DMFs 1099_01 and 5000_17 along with MspI/HpaII CCGG motifs were found upstream a *S*. *spontaneum* transcript assigned to the GO term “chromatin remodeling” and near the cis-regulatory element AT-Hook. Described as “Putative lysine-specific demethylase JMJ14” and possessing the GO term “histone demethylase activity (H3-trimethyl-K4 specific)” from “molecular function” category (GO:0034647), the maize protein A0A1D6HR86 may represent a Jumonji C domain-containing protein 14 (JMJ14). Post-translational modifications of histones are epigenetic markers consisting on covalent addition of different chemical groups to particular residues, resulting in acetylation, methylation, phosphorylation, ubiquitination, or glycosylation; most of them occur in the tails of histones, leading to chromatin remodeling [71]. Jumonji C demethylases are responsible for the removal of several histone methylation markers, e.g., H3K4me2/3, H3K27me3, and H3K9m1/2/3, and have been associated with defense responses in rice and Arabidopsis [72, 73]. Accordingly, the presence of the cis-regulatory sequence AT-Hook within DMFs 1099_01 and 5000_17 reinforces their association with epigenetic pathways, because of their putative functions in DNA methylation and chromatin remodeling [74].

Thus, the *in silico* analysis of DMFs indicated that the changes in cytosine methylation in sugarcane infected with SCMV were distributed within the gene body and heterochromatic regions of transcripts and within genomic regions, in which DMFs were found upstream of the 5′UTR of transcripts and comprised cis-acting elements. The assigned functions of the transcripts included pathways related to plant immunity and other stress responses, epigenetic changes, and TE activity. The cis-acting elements, in turn, either supported these functions or provided additional understanding of the pathways putatively involved with the changes in cytosine methylation.

### Sequencing of DTFs

Sixteen DTFs were reamplified, cloned, and sequenced, ranging from 27 to 211 bp after adapter trimming (Table 4, S1 File). Fifteen DTFs showed significant alignments with transcripts by using BLASTN, all of which were within exons, i.e., 5′-UTR, CDS, or 3′-UTR.

**Table 4:** BLASTN analysis of 16 differentially transcribed fragments (DTFs) and BLASTX analysis of the respective sugarcane transcripts.

DTF (Time points/Selective combination)	Size (bp)	Transcript (Ident/Query cover/e-value/Location)^a^	Uniprot (Species/Annotation score/Ident/Query cover/e-value)^f^	Annotation (Accession)
Upregulaion (s.i)				
5000_20 (24 h/ *Eco*RIAAC/*Mse*ICGT)	63	SP803280_c77422_g1_i1^b^ (98.41/100.00/6e-30/Exon)	A0A0D3CY91 (*Brassica oleracea*/1/28.85/44.00/9e-04)	
5000_21 (24 h/ *Eco*RIACG/*Msp*ITTG)	36	SCRFAM1027D10.g^c^ (100.00/88.00/2e-10/CDS)	A0A3L6DH48 (*Zea mays*/1/92.68/56.00/7.0e-65)	Kelch motif (PF01344)^g^
5000_22 (24 h/ *Eco*RIACG/*Msp*IACT)	29	Sh_219I15_g000030^d^ (96.30/93.00/3e-5/CDS)	Q6ENT5-1 (*Saccharum officinarum*/3/94.50/98.00/2.4e-40)	protein stabilization (GO:0050821)^h^
5000_23 (24 h/ *Eco*RIAAA/*Mse*ICTT)	52	Sh_005D21_g000060^d^ (92.50/100.00/2e-9/3'-UTR)	Q2QVG9 (*Oryza sativa*/2/95.41/46.00/0.0)	protein metabolic process (GO:0019538)^h^
5000_24 (72 h/ *Eco*RIAAA/*Mse*ICTT)	111	Sh_005D21_g000060^d^ (100.00/100.00/1e-47/3'-UTR)	Q2QVG9 (*Oryza sativa*/2/95.41/46.00/0.0)	protein metabolic process (GO:0019538)^h^
5000_25 (24 h/ *Eco*RIAAA/*Mse*ICTT)	51	Sh_005D21_g00006^d^ (94.74/100.00/5e-10/3'-UTR)	Q2QVG9 (*Oryza sativa*/2/95.41/46.00/0.0)	protein metabolic process (GO:0019538)^h^
5000_26 (24 h/ *Eco*RIACG/*Msp*IACT)	139	Sh_083B09_g000030^d^ (97.84/100.00/3e-63/3'-UTR)	A0A0E0AQS3 (*Oryza sativa*/1/98.28/27.00/2.0e-59)	Oxidoreductase family, NAD-binding Rossmann fold; Oxidoreductase family, C-terminal alpha/beta domain (PF01408/PF02894)^g^
5000_27 (24 h/ *Eco*RIACG/*Msp*IACT)	105	SCSP803280_000040222^b^ (86.67/100.00/8e-33/Genomic)		
5000_28 (48 h/ *Eco*RIAGC/*Msp*IACA)	51	SCSBFL1041H02.g^c^ (98.04/100.00/9e-23/5'-UTR)	A0A1D6EE50 (*Zea mays*/1/96.23/26.00/9.0e-35)	metal ion transport (GO:0030001)^h^
5000_29 (48 h/ *Eco*RIACC/*Mse*ICTT)	138	Sh_250G13_g000040^d^ (99.27/99.00/1e-63/CDS)	C5YXQ2 (*Sorghum bicolor*/1/95.27/10.00/8.0e-97)	Methyl-CpG binding domain; CW-type Zinc Finger (PF01429/PF07496)^g^
5000_30 (72 h/ *Eco*RIACA/*Mse*ICTT)	211	SP803280_c102182_g1_i2^b^ (99.52/99.00/4e-103/Exon)	A0A0A8ZSQ5 (*Arundo donax*/1/57.14/11.00/1e-07)	No annotation
5000_31 (72 h/ *Eco*RIACG/*Msp*IACT)	27	comp85702_c0_seq1^e^ (100.00/92.00/2e-10/CDS)	P15804 (*Sorghum bicolor*/4/95.87/96.00/0.0)	tricarboxylic acid cycle (GO:0006099)^h^
1099_32 (24 h/ *Eco*RIAGC/*Msp*IACA)	51	SCSBFL1041H02.g^c^ (96.08/100.00/2e-18/5'-UTR)	A0A1D6EE50 (*Zea mays*/1/96.23/26.00/9.0e-35)	metal ion transport (GO:0030001)^h^
1099_33 (48 h/ *Eco*RIAGC/*Msp*IACA)	51	SCSBFL1041H02.g^c^ (96.08/100.00/2e-19/5'-UTR)	A0A1D6EE50 (*Zea mays*/1/96.23/26.00/9.0e-35)	metal ion transport (GO:0030001)^h^
Downregulation (m.i)				
5000_34 (24 h/ *Eco*RIAAA/*Mse*ICTT)	39	Sh_213J23_g000110^d^ (92.31/100.00/9e-10/CDS)	A0A1D6IPX9 (*Zea mays*/1/81.82/77.00/8.0e-141)	lipid transport (GO:0006869)^h^
5000_35 (24 h/ *Eco*RIACG/*Msp*IACT)	27	comp85702_c0_seq1^e^ (100.00/92.00/2e-10/CDS)	P15804 (*Sorghum bicolor*/4/95.87/96.00/0.0)	tricarboxylic acid cycle (GO:0006099)^h^

^a^: BLASTN alignment between transcript and genomic clusters of sugarcane.

^b^: Hits with long-read libraries of SP80-3280 from CTBE database.

^c^: Hits with expressed sequence tags (ESTs) of SP80-3280 from SUCEST-FUN database.

^d^: Hits with mosaic monoploid reference of R570 from CIRAD database.

^e^: Hits with gene space assembly of SP80-3280 from NCBI database.

^f^: BLASTX alignment between sugarcane transcript and proteins from Uniprot database.

^g, h^: Pfam motifs and Gene Ontology (GO) terms from the "Biological Process" category, respectively, addressed to the proteins from Uniprot database.

DTF 5000_29 aligned to the transcript Sh_250G13_g000040, which is described in the CIRAD database as “methyl-binding domain protein MBD101” and was assigned to the pfam motifs “zinc finger CW type” (zf-CW) and “methyl CpG DNA-binding domain” (MBD). The putative function of MBD motif is to recognize and bind to methylated DNA, possibly acting as the mediator of heterochromatin formation [75].

Changes in the transcription of F-box proteins are suggested for the transcript SCRFAM1027D10.g, aligned to DTF 5000_21 by the BLASTX analysis, which revealed significant alignment with the maize protein A0A3L6DH48, described as “F-box/kelch-repeat protein SKIP11.” F-box proteins are known to be associated with varied functions, including defense against pathogens and chromatin remodeling [76].

Putative associations to changes in the transcriptome of photosynthesis pathways were observed for DTFs 5000_22, 5000_31, and 5000_35. DTF 5000_22 aligned to the sugarcane transcript Sh_219I15_g000030, described as “Photosystem II reaction center protein H,” was assigned to the GO term “protein stabilization”. Transcripts associated with Photosystem II were also differentially regulated in potato infected with potato virus Y (PVY) [77] and in pea plants infected with plum pox virus [78]. The RNA-seq transcript comp85702_c0_seq1, aligned to our DTFs 5000_31 and 5000_35, is similar to the sorghum protein P15804, described as “Phosphoenolpyruvate carboxylase 3” (PEPCase 3), also carrying the GO term “phosphoenolpyruvate carboxylase activity” from the “molecular function” category (GO:0008964). Likewise, changes in PEPCase gene expression have been reported in tobacco plants infected with potyviruses, i.e. potato virus A and PVY [79].

The alignment analysis of DTFs also revealed that SCMV infection might change the transcriptional levels of oxidoreductases and synaptotagmin (SYT) proteins. The former was revealed by the alignment between DTF 5000_26 and transcript Sh_083B09_g000030, which is described as “similar to Organic cation transporter-like protein” and assigned to the pfam motifs described as “Oxidoreductase family, NAD-binding Rossmann fold.” Similarly, oxidoreductases have been shown to be activated in *Arabidopsis thaliana* in response to turnip mosaic virus infection by a genome-wide association study [80]. Alignment with SYT proteins and the GO term “lipid transport” was observed for DTF 5000_34, which was downregulated according to cDNA-AFLP observations. The SYTA protein is the best studied among *A*. *thaliana* SYT proteins, which play a role in the response to abiotic and biotic stresses [81]. Uchiyama et al. [82] reported that SYTA regulates the intercellular movement of plant viruses cabbage leaf curl virus, tobacco mosaic virus, and turnip mosaic virus.

Changes in the transcriptome of sugarcane challenged with SCMV infection may also involve molecular chaperones. Such association was observed for DTFs 5000_23, 5000_24, and 5000_25, which aligned with the sugarcane transcript Sh_005D21_g000060 and the *Oryza sativa* protein Q2QVG9, both described as “Chaperone protein ClpC2, chloroplastic.” The pfam motifs present in Q2QVG9 are described as molecular chaperones “caseinolytic protease” (Clp) of “Heat Shock Protein 100” (Hsp100) class and “ATPases associated with diverse cellular activities” (AAA). Molecular chaperones enable the proper folding of newly synthesized proteins, preventing protein aggregation and assisting in the folding of multiprotein complexes. Conversely, AAA proteases degrade irreparably damaged proteins or erroneously synthesized proteins [83]. Another putative association with molecular chaperones was observed for DTFs 5000_28, 1099_32, and 1099_33, which aligned to the SUCEST transcript SCSBFL1041H02.g and were assigned to the GO term “metal ion transport” and the pfam motif “heavy metal-associated” (HMA). This suggests changes in the gene expression of “heavy metal-associated isoprenylated plant proteins” (HIPPs), defined as metallochaperones with one or two HMA domains [84]. The HIPPS proteins have been reported as regulatory elements in the transcriptional responses to abiotic stresses such as cold and drought [85].

PlantPAN analysis revealed cis-regulatory sequences upstream the TSS for all DTFs assigned to GO annotations. CpG islands were found to comprise the TSS for the transcripts aligned with DTFs 5000_21, 5000_29, 5000_31, and 5000_35. The transcripts aligned to DTFs 5000_23 and 5000_26 showed CpG islands located less than 1000 bp upstream and/or downstream the TSS, whereas close tandem repeat was observed for 5000_29. However, no close CpG islands or tandem repeats were observed for 5000_22 and 5000_34 (S4 Table). The transcripts associated with DTFs 5000_28, 1099_32, and 1099_33 did not align with known sugarcane genomic sequences; therefore, putative cis-acting sequences and the proximity of CpG islands could not be assessed.

The cis-regulatory sequence bZIP, observed for the transcripts aligned to DTFs 5000_26 and 5000_34, along with the sequence TALE, associated with DTFs 5000_23, 5000_24, and 5000_25, further indicated the association with stress responses. For instance, the presence of stress-responsive bZIP transcription factor-binding site is in accordance with the annotations assigned to the sugarcane transcripts Sh_083B09_g000030 and Sh_213J23_g000110, respectively, reported as responsive to potyvirus infection and as an intermediate of intercellular movement of potyviruses [80, 82]. Moreover, the cis-regulatory sequence TALE includes transcriptional activators of plant genes during pathogenesis [86], which indicates response to biotic stresses for the transcript Sh_005D21_g000060, assigned to molecular chaperones and protein quality control [83]. The cis-acting element (E2F; E2F/DP) of the comp85702_c0_seq1 transcript, aligned to DTFs 5000_31 and 5000_35, corresponds to the E2F transcription factor, which plays a key role in plant growth and development by regulating cell-cycle-related genes and some plant-specific pathways such as photosynthesis [87].

The alignments of DTFs revealed transcripts associated with a diverse range of pathways, some of which involved proteins reported to have associations with potyvirus infection, i.e., photosynthesis, oxidoreductases, and lipid transport, whereas the remaining DTFs were assigned to epigenetic changes, defense against pathogens, protein quality control, and responses to abiotic stresses. The association of changes in the transcriptome detected using cDNA-AFLP with epigenetic pathways is more evident for DTF 5000_29, based on the assigned annotations and TSS alignment within a CpG island (Table 4 and S4 Table). The transcriptional regulation of other DTFs may also be associated with epigenetic pathways, because of the TSS alignments within CpG islands, i.e., 5000_21, 5000_31, and 5000_35, or those located at less than 1 Kb downstream, i.e., 5000_23, 5000_24, 5000_25, and 5000_26. Another indication is the presence of the cis-regulatory sequences AT-Hook and Homeodomain; ZF-HD associated with DNA methylation and chromatin remodeling, respectively, observed for transcripts aligned to DTFs 5000_21 and 5000_22 (S4 Table).

### Expression analysis

We evaluated the transcript accumulation of genes aligned to DTFs 5000_24 and 5000_29 as well as to DMFs 5000_06 and 5000_14 (Tables 3 and 4). We also evaluated the transcript SCQGST1032C04.g, retrieved from SUCEST database via alignment with a DTF described by Medeiros et al. [15], which refers to proteins from the VQ motif family; it is upregulated at 72 hpi according to cDNA-AFLP analysis. The primer sequences, amplicon sizes, RT-qPCR efficiencies, and correlation coefficients (R^2^) for the four primer pairs designed are listed in S5 Table.

**Fig 2:**
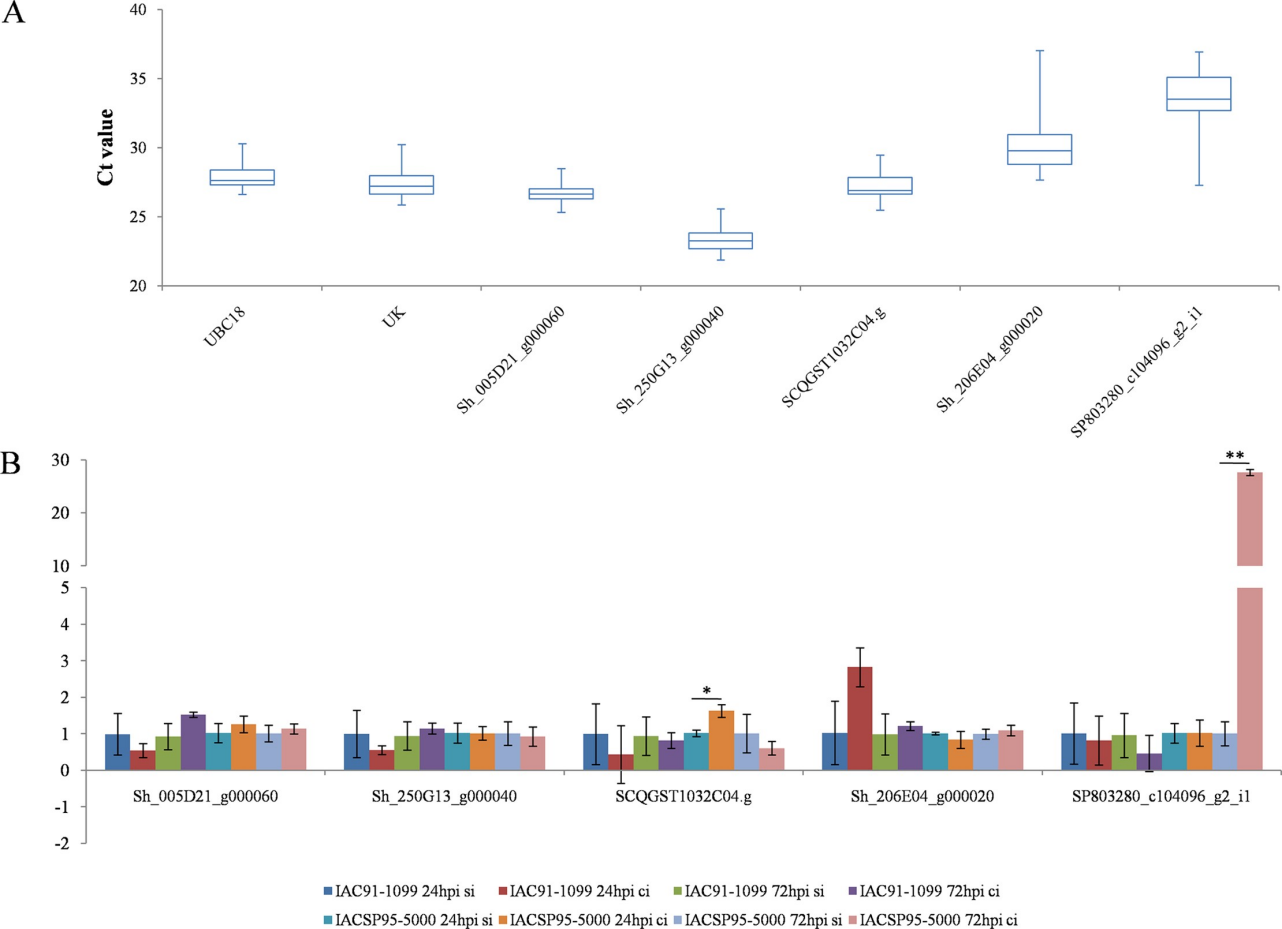
a) Box-plot representing the distribution of the 72 raw Ct values for each gene. The whiskers denote the highest and lowest Ct values, whereas the lower and upper boundaries of the box (interquartile) represent the 25^th^ and 75^th^ percentile, respectively. The mean values of each gene are represented by the line within the boxes. b) Average relative normalized expression of the three transcripts aligned to differentially transcribed fragments (DTFs), i.e., Sh_005D21_g0060, Sh_250G13_g000040, and SCQGST1032C04.g, and two transcripts aligned to differentially methylated fragments (DMFs), i.e., Sh_206E04_g000020 and SP803280_c104096_g2_i1, selected based on their assigned annotations from Uniprot. Results represent the fold change in comparison with mock-inoculated samples, normalized to the transcript abundance of ubiquitin-conjugating enzyme 18 (UBC18) and uridylate kinase (UK). Error bars indicate standard error of the mean (n = 3 biological replicates from a single experiment). Asterisks (*p< 0.05; **p< 0.01) indicate statistically significant differences of the mean values determined using Student’s *t*-test.

According to the standard error of the mean, calculated from the log-transformed normalized expression [40], the tested genes showed variations among the three biological replicates (Fig 2; S2 Appendix). Despite these variations, we found that the SCQGST1032C04.g gene was upregulated at 24 hpi by the factor of 1.60 (*p*< 0.05) and SP803280_c104096_g2_i1 gene was upregulated at 72 hpi by the factor of 27.41 (*p*< 0.01) in the resistant cultivar, IACSP95-5000.

Although DTF 5000_29 was upregulated at 48 hpi, we checked the accumulation of Sh_250G13_g000040 transcript in the validation experiment, considering its possible role in the changes in cytosine methylation observed using the MSAP analysis. This transcript did not seem to be involved in the changes in cytosine methylation at 24 and 72 hpi, since the fold changes were not significant according to Student’s two-tailed *t*-test for unpaired observations. In the remaining two DTFs, RT-qPCR results diverged from previous cDNA-AFLP observations, i.e., significant upregulation of SCQGST1032C04.g at 24 hpi instead of at 72 hpi and non-significant changes in the expression levels of the transcript Sh_005D21_g000060.

The findings of methods used for analyzing the transcriptome, such as RNA-seq and cDNA-AFLP, usually correlate well with those of RT-qPCR [88, 89]. The disagreements between transcriptome analyses and RT-qPCR could be associated with the differences in sensitivity inherent to the techniques [90], low gene expression levels [88] and presence of different putative paralogues in the genome [91]. RT-qPCR results revealed low gene expression, which may explain the low similarity with the cDNA-AFLP results. Conversely, the likely influence of paralogues is particularly relevant considering the hybridization and polyploidization events that occurred during the sugarcane breeding process and the reports on paralogous gene duplications in the sugarcane genome [92, 93]. Nonetheless, the significant upregulation of SCQGST1032C04.g at 24 hpi in the resistant cultivar, IACSP95-5000, indicated the involvement of VQ proteins in the genetic resistance of sugarcane to SCMV. The VQ proteins regulate diverse developmental processes and responses to abiotic and biotic stresses [94]; they have been shown to be involved in the association between defense signaling and chloroplast function [95].

The RT-qPCR results also indicated that the changes in cytosine methylation at the intronic regions of Sh_206E04_g000020, i.e., DMF 5000_06, putatively associated with PTI pathways, did not significantly change the gene expression. Conversely, the hypermethylation event that occurred at 24 hpi at 3′ UTR of SP803280_c104096_g2_i1, i.e., DMF 5000_14, putatively associated with proteolysis pathways, could play a role in the observed upregulation of this transcript at 72 hpi. This result differs from that reported by Abid et al. [28], who showed negative correlations between cytosine methylation and gene expression. Such trend has commonly been observed in other MSAP studies; however, instances of transcript upregulation in association with hypermethylation events, more specifically at the 3′-UTR, have also been reported [96, 97]. The effects of cytosine methylation at gene-body regions on gene expression are not yet well understood [58]; nonetheless, according to Li et al. [98], they usually contrast with the effects of cytosine methylation at the promoter regions and are generally positively correlated with gene expression. However, this correlation is complex since it depends on the methylation level, with the highest expression levels noted for genes with moderate gene-body methylation, while heavy gene-body methylation seems to repress gene expression [98]. The differences between the time of change in cytosine methylation and that in the expression level of the associated transcript may represent its transition toward a moderate level of gene-body methylation. Similarly, cytosine hypomethylation of the gene encoding glycerophosphodiesterase-like protein preceded the transcript upregulation by 5h in *Nicotiana tabacum* under abiotic stresses [99].

## Conclusions

The results of MSAP patterns, DMF alignments, and RT-qPCR provided important information regarding the changes in cytosine methylation in sugarcane submitted to SCMV infection, revealing its extension, putative distribution, biological relevance, and effects on gene expression. The involvement of epigenetic pathways is reinforced by the DTF alignments, whereas the RT-qPCR results confirmed the upregulation of one candidate gene identified by cDNA-AFLP. According to Nicaise [50], the currently known antiviral immune mechanisms in plants are nucleotide-binding site–leucine-rich repeat dominant resistance, recessive resistance, RNAi, hormone-mediated resistance, and PTI pathways, many of which corroborate with our results. Accordingly, this study provides potential candidate genes capable of revealing the molecular processes underlying genetic resistance of sugarcane to SCMV.

## Supporting information

S1 Table MSAP (EcoRI/HpaII-MspI) selective primer combinations. The three base selective nucleotides are presented in bold.
(DOC)

S2 Table Promoter analysis for the assessment of putative regulatory elements of transcripts aligned to DMFs by PlantPAN. (DOCX)

S3 Table PlantPAN promoter analysis for the assessment of putative regulatory elements of DMFs aligned to genomic regions. (DOCX)

S4 Table PlantPAN promoter analysis for the assessment of putative regulatory elements of transcripts aligned to DTFs. (DOCX)

S5 Table Forward (FW) and reverse (RV) primer pairs sequences, amplicon size (A) in base pairs (bp), melting temperature (Tm), PCR reaction efficiency (E), and coefficient of determination (R2) of reference genes and candidate genes selected for validation via RT-qPCR.
(DOC)

S1 Fig MSAP molecular profile of the 24 sugarcane leaf samples using the selective combination EcoRIaca (IR700) and HpaII/MspIacc. PCR products were separated in 6% denaturing polyacrylamide gel. The arrow indicates a differentially methylated fragment (DMF).
(DOC)

S1 File Sequences of DMFs and DTFs. (TXT)

S2 File (PDF)

S1 Appendix Raw MSAP data, with presence (1) or absence (0) scores for each of the 1131 loci across the 24 sugarcane leaf samples and 29 selective primer combinations. (XLSX)

S2 Appendix Ct values of the reference genes and the candidate genes used for the RT-qPCR validation. (XLSX)
