# Supplementary material for: Sugarcane mosaic virus mediated changes in cytosine methylation pattern and differentially transcribed fragments in resistance-contrasting sugarcane genotypes
Source: PLoS One. 2020 Nov 9;15(11):e0241493. doi: 10.1371/journal.pone.0241493 (PMC7652275; doi:10.1371/journal.pone.0241493)
Supplement: S1 Table — The three base selective nucleotides are presented in bold. (DOC) [file pone.0241493.s001.doc]

S1 Table. MSAP (EcoRI/HpaII-MspI) selective primer combinations. The three base selective nucleotides are presented in bold.

| Sequence (5'-3') | Sequence (5'-3') |
| --- | --- |
| CTGCGTACCAATTC**aca**/GATGAGTCCTGATCGG**ttg** | CTGCGTACCAATTC**aca**/GATGAGTCCTGATCGG**act** |
| CTGCGTACCAATTC**aca**/GATGAGTCCTGATCGG**acc** | CTGCGTACCAATTC**aca**/GATGAGTCCTGATCGG**gaa** |
| CTGCGTACCAATTC**aga**/GATGAGTCCTGATCGG**ttg** | CTGCGTACCAATTC**acc**/GATGAGTCCTGATCGG**aca** |
| CTGCGTACCAATTC**aga**/GATGAGTCCTGATCGG**acc** | CTGCGTACCAATTC**aga**/GATGAGTCCTGATCGG**gaa** |
| CTGCGTACCAATTC**aca**/GATGAGTCCTGATCGG**tcg** | CTGCGTACCAATTC**agg**/GATGAGTCCTGATCGG**aca** |
| CTGCGTACCAATTC**aca**/GATGAGTCCTGATCGG**tag** | CTGCGTACCAATTC**acc**/GATGAGTCCTGATCGG**ttg** |
| CTGCGTACCAATTC**aga**/GATGAGTCCTGATCGG**tcg** | CTGCGTACCAATTC**acc**/GATGAGTCCTGATCGG**acc** |
| CTGCGTACCAATTC**aga**/GATGAGTCCTGATCGG**tag** | CTGCGTACCAATTC**acc**/GATGAGTCCTGATCGG**tcg** |
| CTGCGTACCAATTC**aca**/GATGAGTCCTGATCGG**cgc** | CTGCGTACCAATTC**agg**/GATGAGTCCTGATCGG**ttg** |
| CTGCGTACCAATTC**aca**/GATGAGTCCTGATCGG**gag** | CTGCGTACCAATTC**acc**/GATGAGTCCTGATCGG**cgc** |
| CTGCGTACCAATTC**aga**/GATGAGTCCTGATCGG**cgc** | CTGCGTACCAATTC**agg**/GATGAGTCCTGATCGG**tcg** |
| CTGCGTACCAATTC**aga**/GATGAGTCCTGATCGG**gag** | CTGCGTACCAATTC**agg**/GATGAGTCCTGATCGG**acc** |
| CTGCGTACCAATTC**aca**/GATGAGTCCTGATCGG**aca** | CTGCGTACCAATTC**acc**/GATGAGTCCTGATCGG**tag** |
| CTGCGTACCAATTC**aga**/GATGAGTCCTGATCGG**act** | CTGCGTACCAATTC**agg**/GATGAGTCCTGATCGG**tag** |
| CTGCGTACCAATTC**aga**/GATGAGTCCTGATCGG**aca** |  |
