# Supplementary material for: Sugarcane mosaic virus mediated changes in cytosine methylation pattern and differentially transcribed fragments in resistance-contrasting sugarcane genotypes
Source: PLoS One. 2020 Nov 9;15(11):e0241493. doi: 10.1371/journal.pone.0241493 (PMC7652275; doi:10.1371/journal.pone.0241493)
Supplement: S2 Table — (DOCX) [file pone.0241493.s002.docx]

S2 Table. Promoter analysis for the assessment of putative regulatory elements of transcripts aligned to DMFs by PlantPAN.

| Genomic cluster | Transcript  (%ID/query cover/e-value)^a^ | Annotation  (Accession) | DMF  (Start/end)^h^  CCGG  (Start/end)^h^ | Site name  (Score/Sequence)  (Position/Strand)^h^ | CpG island^h^ | Tandem repeat^h^ |
| --- | --- | --- | --- | --- | --- | --- |
| Sh04 | Sh_018M23_contig-1_g000070^b^  (100.0/100.0/0.0) | mRNA splicing, via spliceosome  (GO:0000398)^e^ | 1099_03  (-706/-663)  (-710/-707) | bZIP  (0.95/cctaaCACGTtat)  (-33/-) | -4078/-3074  -1675/-281 |  |
| CM010714.1 | Sspon.08G0008750-3D^c^  (100.0/100.0/0.0) | Myb-like DNA-binding domain (PF00249)^f^ | 5000_05  (200/284)  (1344/1347) | Homeodomain; ZF-HD  (1/ctTAATCtaa)  (-12/+) |  | -2788/-2753  475/499 |
| Sh04 | Sh_206E04_g000020^b^  (100.0/100.0/0.0) | protein phosphorylation  (GO:0006468)^e^ | 5000_06  (2190/2281)  (2185/2188) | AP2; ERF  (1/gCGCCGgc)  (-60/-) | -3272/-1412  -944/2691 | -2245/-1807  1473/1519  1789/1821  1793/1833  2196/2220 |
| CM010705.1 | Sspon.06G0001250-2C^c^  (100.0/100.0/0.0) | cell surface receptor signaling pathway  (GO:0007166)^e^ | 5000_09  (25161/25164) | EIN3; EIL  (0.99/atATGCAtgg)  (-14/+) | -2333/-959  -282/623  4812/5744  9177/12796  14817/16446  20267/26494 | -1444/-1397  -1436/-1392  5212/5276  5798/5855  5817/6102  19163/19209 |
| CM010688.1 | Sspon.02G0041100-1B^c^  (100.0/100.0/0.0) |  | 5000_10  (-1086/-981)  (-1181/-1178) | Myb/SANT; MYB  (0.97/cACCGGtg)  (-22/+) | -1674/-649 |  |
| CM010702.1 | Sspon.05G0013670-1P^c^  (99.66/96.00/0.0) | DNA binding  (GO:0003677)^g^ | 1099_11  (2254/2413)  (2250/2253) | bZIP  (0.97/tGACAGatct)  (-13/+) | -1088/-589  344/1041  9518/10145  11085/12713  15877/17683 | -2142/-2105  -1911/-1865  -593/-548  612/669  18827/18866 |
| Sh10 | Sh_241P15_contig-1_g000060^b^  (100.0/100.0/0.0) | oligopeptide transmembrane transport (GO:0035672)^e^ | 5000_13  (943/1086)  (1087/1090) | bZIP  (0.98/ tgcTGACGtac)  (-1/-) | -426/2357 |  |
| SCSP803280_000102643 | SP803280_c104096_g2_i1^d^  (99.57/99.00/0.0) | protein catabolic process  (GO:0030163)^e^ | 5000_14  (1605/1758)  (1601/1604) | EIN3; EIL  (0.98/aagATACAc)  (-13/-) | -196/1967  2637/3315 |  |
| SCSP803280_000116158 | SP803280_c132337_g1_i1^d^  (81.00/71.02/3e-63) | Reverse transcriptase; Reverse transcriptase-like (PF00078 / PF13456)^f^ | 5000_16  (176/385)  (-124/-121) | bZIP  (0.75/ACACGgga)  (-22/+) | -350/538 |  |
| SCSP803280_000000889 | SP803280_c89867_g1_i4^d^  (99.88/77.00/0.0) | NB-ARC domain (PF00931)^f^ | 5000_18  (-176/-61)  (-60/-57) | NAC; NAM  (0.98/ctaGCGTAaa)  (-3/+) | -3451/-1856  -1563/-361  33/755 |  |

^a^: BLASTN alignment between transcript and genomic clusters of sugarcane. ^b^: Sequences of mosaic monoploid reference of R570 from CIRAD database; ^c^: Sequences of *S. spontaneum* AP85-441 haploid assembly; ^d^: Sequences of long-read libraries of SP80-3280 from CTBE database. ^e, f, g^: Gene Ontology (GO) terms from the "Biological Process" category, Pfam motifs, and GO terms from the "Molecular function" category, respectively, from proteins from Uniprot database. ^h^: Relative position to the transcriptional start site (TSS).
