# Supplementary material for: Sugarcane mosaic virus mediated changes in cytosine methylation pattern and differentially transcribed fragments in resistance-contrasting sugarcane genotypes
Source: PLoS One. 2020 Nov 9;15(11):e0241493. doi: 10.1371/journal.pone.0241493 (PMC7652275; doi:10.1371/journal.pone.0241493)
Supplement: S3 Table — (DOCX) [file pone.0241493.s003.docx]

S3 Table. PlantPAN promoter analysis for the assessment of putative regulatory elements of DMFs aligned to genomic regions.

| Genomic cluster | (Downstream transcript/%ID/query cover/e-value)^a^  (Protein ID/Species/ Annotation score /Ident/Query cover/e-value)^b^ |  | DMF  (Start/end)^i^  CCGG  (Start/end) | Site name  (Score/Sequence)^i^  (Position/Strand) | CpG island^g^ | Tandem repeat^i^ |
| --- | --- | --- | --- | --- | --- | --- |
| Sh02 | (Sh_251N21_g000080/72.76/3.0/9.0e-31)^c^  (A0A194YQU0/ *Sorghum bicolor*/1/73.18/10.0/5e-67) | No annotation | 1099_01  (-2349/-2267)  (-2424/-2421) | AT-Hook  (1/cagaAAAAA)  (-2276/+) | -4967/-2889  -1153/734 | -37/254  55/115  576/611 |
| SCSP803280_000000185^b^ | (SP803280_c97920_g2_i1/81.0/32.0/9e-28)^d^  (A0A368RWK0/ *Setaria italica*/1/35.71/88.0/4e-9) | No annotation | 1099_01  (No TSS)* |  |  |  |
| CM010688.1^c^ | (Sspon.02G0043430-1P/95.02/64.0/0.0)^e^  (A0A1D6HR86/ *Zea mays*/2/79.65/58.00/9e-134) | chromatin remodeling (GO:0006338)^f^ | 1099_01  (-1079/-998)  (-926/-923) | AT-Hook  (1/TTTTTtctg)  (-1061/-) | -3675/-3624  -3379/-2133  -1047/582 | -3675/-3624 |
| SCSP803280_000016069^b^ | (SP803280_c78142_g1_i2/98.89/100.00/0.0)^d^  (A0A1W0VYA9/ *Sorghum bicolor*/1/55.00/16.0/7e-11) | No annotation | 1099_04  (No TSS)* |  |  |  |
| CM010698.1^c^ | (Sspon.02G0044550-1B /6.00/73.47/7e-35)^e^  (A0A1W0VYA9/ *Sorghum bicolor*/1/100.00/23.00/6e-117) | No annotation | 1099_04  (-649/-533)  (-532/-529) | (Motif sequence only)  (gGTTGG)  (-531/-) | -3652/-2409  189/1983 | 840/871 |
| Sh02 | (Sh_251N21_g000080/72.76/3.0/9.0e-31)^c^  (A0A194YQU0/ *Sorghum bicolor*/1/73.18/10.0/5e-67) | No annotation | 5000_17  (-2349/-2267)  (-2421/-2424) | AT-Hook  (1/cagaAAAAA)  (-2277/+) | -4967/-2889  -1153/734 | -37/254  55/115  576/611 |
| CM010688.1 | (Sspon.02G0043430-1P/95.02/64.0/0.0)^e^  (A0A1D6HR86/ *Zea mays*/2/79.65/58.00/9e-134) | chromatin remodeling (GO:0006338)^f^ | 5000_17  (-1079/-998)  (-926/-923) | AT-Hook  (TTTTTtctg)  (-1061/-) | -3444/-2198  -1051/517 | -3675/-3624 |
| SCSP803280_000016069^b^ | (SP803280_c78142_g1_i2/98.89/100.00/0.0)^d^  (A0A1W0VYA9/ *Sorghum bicolor*/1/55.00/16.0/7e-11) | No annotation | 5000_19  (No TSS)* |  |  |  |
| CM010698.1^c^ | (Sspon.02G0044550-1B/6.00/73.47/7e-35)^e^  (A0A1W0VYA9/ *Sorghum bicolor*/1/100.00/23.00/6e-117) | No annotation | 5000_19  (-649/-533)  (-532/-529) | (Motif sequence only)  (gGTTGG)  (-531/-) | -3652/-2409  189/1983 | 840/871 |

^a^: BLASTN alignment between transcript and genomic clusters of sugarcane. ^b^: BLASTX alignment between sugarcane transcript and proteins of maize and sorghum; ^c^: Hits with transcripts from the mosaic monoploid reference of R570 from CIRAD database. ^d^: Hits with transcripts from the long-read libraries of SP80-3280 from CTBE database. ^e^: Hits with transcripts from the *S. spontaneum* AP85-441 haploid assembly. ^f^: Gene Ontology (GO) terms from the "Biological Process" category addressed to the proteins from Uniprot database. ^g^: Relative position to the transcriptional start site (TSS).
