# Supplementary material for: Sugarcane mosaic virus mediated changes in cytosine methylation pattern and differentially transcribed fragments in resistance-contrasting sugarcane genotypes
Source: PLoS One. 2020 Nov 9;15(11):e0241493. doi: 10.1371/journal.pone.0241493 (PMC7652275; doi:10.1371/journal.pone.0241493)
Supplement: S4 Table — (DOCX) [file pone.0241493.s004.docx]

S4 Table. PlantPAN promoter analysis for the assessment of putative regulatory elements of transcripts aligned to DTFs.

| Genomic cluster | Transcript  (%ID/query cover/e-value)^a^ | Annotation  (Accession) | DTF (Start/end)^g^ | Site name  (Sequence)  (Position/Strand)^g^ | CpG island^g^ | Tandem repeat^g^ |
| --- | --- | --- | --- | --- | --- | --- |
| SCSP803280_000171265 | SCRFAM1027D10.g^b^  (99.50/100.00/0.0) | Kelch motif (PF01344)^e^ | 5000_21  (243/274) | AT-Hook  (TTTTTgggg)  (-16/-) | -1597/369 |  |
| Sh04 | Sh_219I15_g000030^c^  (100.00/100.00/8e-116) | protein stabilization  (GO:0050821)^f^ | 5000_22  (49/75) | Homeodomain; ZF-HD  (aatGATTAaa)  (-18/-) |  |  |
| Sh08 | Sh_005D21_g000060^c^  (100.00/100.00/0.0) | protein metabolic process  (GO:0019538)^f^ | 5000_23  (5797/5855)  5000_24  (5712/5815)  5000_25  (5800/5855) | TALE  (TGACC)  (-5/+) | -923/-320  16/573  5008/5852 | 1134/1174 |
| Sh07 | Sh_083B09_g000030^c^  (100.00/100.00/0.0) | Oxidoreductase family, NAD-binding Rossmann fold; Oxidoreductase family, C-terminal alpha/beta domain (PF01408/PF02894)^e^ | 5000_26  (3114/3252) | bZIP  (ACAGGtat)  (-8/+) | -2408/-1896  -1603/-573 |  |
|  |  |  |  |  |  |  |
| Sh09 | Sh_250G13_g000040^c^  (100.00/100.00/0.0) | Methyl-CpG binding domain; CW-type Zinc Finger  (PF01429/PF07496)^e^ | 5000_29  (8064/8200) | AP2; ERF  (cCGCCGgcat)  (-8/+) | -2851/-1796  -369/426  2347/7345 | -867/-843  4056/4088  4304/4432 |
| QPEU01370577.1 | comp85702_c0_seq1^d^  (99.70/100.00/0.0) | tricarboxylic acid cycle  (GO:0006099)^f^ | 5000_31; 5000_35  (6857/6880) | E2F; E2F/DP  (gcggGCGCGg)  (-2/-) | 4712/6926  -899/988 | -759/-705 |
| Sh02 | Sh_213J23_g000110^c^  (100.00/100.00/0.0) | lipid transport (GO:0006869)^f^ | 5000_34  (374/412) | bZIP  (AAACGagt)  (-25/+) |  |  |

^a^: BLASTN alignment between transcript and genomic clusters of sugarcane. ^b^: Sequences of the expressed sequence tags (ESTs) of SP80-3280 from SUCEST-FUN database. ^c^: Sequences of the mosaic monoploid reference of R570 from CIRAD database. ^d^: Sequences of the gene space assembly of SP80-3280 from NCBI database. ^e, f^: Pfam motifs and Gene Ontology (GO) terms from the "Biological Process" category, respectively, addressed to the proteins from Uniprot database. ^g^: Relative position to the transcriptional start site (TSS).
