# Supplementary material for: Sugarcane mosaic virus mediated changes in cytosine methylation pattern and differentially transcribed fragments in resistance-contrasting sugarcane genotypes
Source: PLoS One. 2020 Nov 9;15(11):e0241493. doi: 10.1371/journal.pone.0241493 (PMC7652275; doi:10.1371/journal.pone.0241493)
Supplement: S5 Table — Forward (FW) and reverse (RV) primer pairs sequences, amplicon size (A) in base pairs (bp), melting temperature (Tm), PCR reaction efficiency (E), and coefficient of determination (R2) of reference genes and candidate genes selected for validation via RT-qPCR. (DOC) [file pone.0241493.s005.doc]

S5 Table. Forward (FW) and reverse (RV) primer pairs sequences, amplicon size (A) in base pairs (bp), melting temperature (Tm), PCR reaction efficiency (E), and coefficient of determination (R^2^) of reference genes and candidate genes selected for validation via RT-qPCR.

| Housekeeping gene ID | Gene accession | Sequence (5’-3’) | A (bp) | Tm (ºC) | E (%) | R^2^ |
| --- | --- | --- | --- | --- | --- | --- |
| UBC18^a^ | SCEZSD1083D06.g^c^ | FW: GCCTGTCAGCCTTCCTTAC  RV: GGTAAGCTTCGCAAATCCAATAG | 100 | 79.1 | 100 | 0.998 |
| UK^a^ | SCQSST1039D09.g^c^ | FW: GCAATCTAAGGGACATAATAAAGGTG  RV: AATCGAATTGCCTACTGATATGTTG | 140 | 77.9 | 100 | 0.984 |
| Fragment ID | Gene accession | Sequence (5’-3’) | A (bp) | Tm (ºC) | E (%) | R^2^ |
| 5000_24 | Sh_005D21_g000060^d^ | FW: CTCGTACATAACAGATGATGACAAA  RV: AAAGGCAGCAACGATTTCC | 123 | 71.2 | 91.4 | 1.000 |
| 5000_29 | Sh_250G13_g000040^d^ | FW: GAAGCAGGAGAAGGCAGAG  RV: GATAGGATGGGACATGATCTTGAG | 100 | 75.8 | 92.9 | 1.000 |
| DTF_14^b^ | SCQGST1032C04.g^c^ | FW: CAACACTGGTACAAGAGACTAGAA  RV: TGTCACCTTCATGGTCATAGC | 83 | 73.7 | 98.6 | 1.000 |
| 5000_06 | Sh_206E04_g000020^d^ | FW: TTGCCCAATTCTCCTATGTG  RV: GCTTGCTACAGATGGAATCA | 100 | 80.3 | 68.2 | 0.977 |
| 5000_14 | SP803280_c104096_g2_i1^e^ | FW: GAATCCTAGGTTGGCTTCCAT  RV: GGTCCACTAGCAGAACATACTC | 81 | 79.5 | 91.9 | 0.998 |

^a^: reference genes described by Silva et al. [39]; ^b^: DTF described by Medeiros et al. [15]; ^c^: expressed sequence tag (EST) of SP80-3280 from SUCEST-FUN database; ^d^: Gene from the mosaic monoploid reference of R570 from CIRAD database; ^e^: Sequence from the long-read libraries of SP80-3280 from CTBE database.
