# Supplementary material for: Sugarcane mosaic virus mediated changes in cytosine methylation pattern and differentially transcribed fragments in resistance-contrasting sugarcane genotypes
Source: PLoS One. 2020 Nov 9;15(11):e0241493. doi: 10.1371/journal.pone.0241493 (PMC7652275; doi:10.1371/journal.pone.0241493)
Supplement: S1 Fig — PCR products were separated in 6% denaturing polyacrylamide gel. The arrow indicates a differentially methylated fragment (DMF). (DOC) [file pone.0241493.s006.doc]

24 hpi m.i MspI

24 hpi m.i MspI

24 hpi m.i HpaII

24 hpi s.i HpaII

48 hpi m.i MspI

48 hpi s.i MspI

48 hpi m.i HpaII

48 hpi s.i HpaII

72 hpi m.i MspI

72 hpi s.i MspI

72 hpi m.i HpaII

72 hpi s.i HpaII

24 hpi s.i MspI

24 hpi m.i HpaII

24 hpis.i HpaII

48 hpi m.i MspI

48 hpi s.i MspI

48 hpi m.i HpaII

48 hpi s.i HpaII

72 hpi m.i MspI

72 hpi s.i MspI

72 hpi m.i HpaII

72 hpi s.i HpaII

IAC91-1099

IACSP95-5000

24 hpi m.i MspI


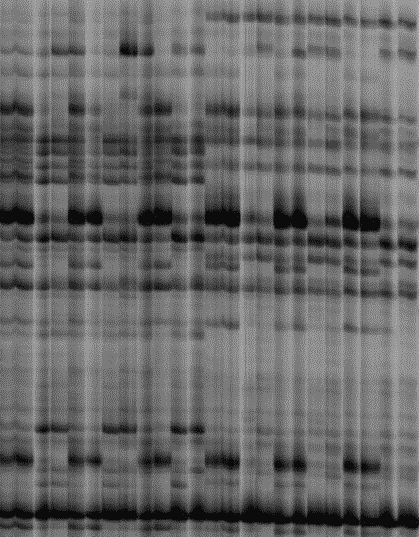


S1 Fig. MSAP molecular profile of the 24 sugarcane leaf samples using the selective combination EcoRIaca (IR700) and HpaII/MspIacc. PCR products were separated in 6% denaturing polyacrylamide gel. The arrow indicates a differentially methylated fragment (DMF).
