# Supplementary material for: Sugarcane mosaic virus mediated changes in cytosine methylation pattern and differentially transcribed fragments in resistance-contrasting sugarcane genotypes
Source: PLoS One. 2020 Nov 9;15(11):e0241493. doi: 10.1371/journal.pone.0241493 (PMC7652275; doi:10.1371/journal.pone.0241493)
Supplement: S2 File — (PDF) [file pone.0241493.s008.pdf]

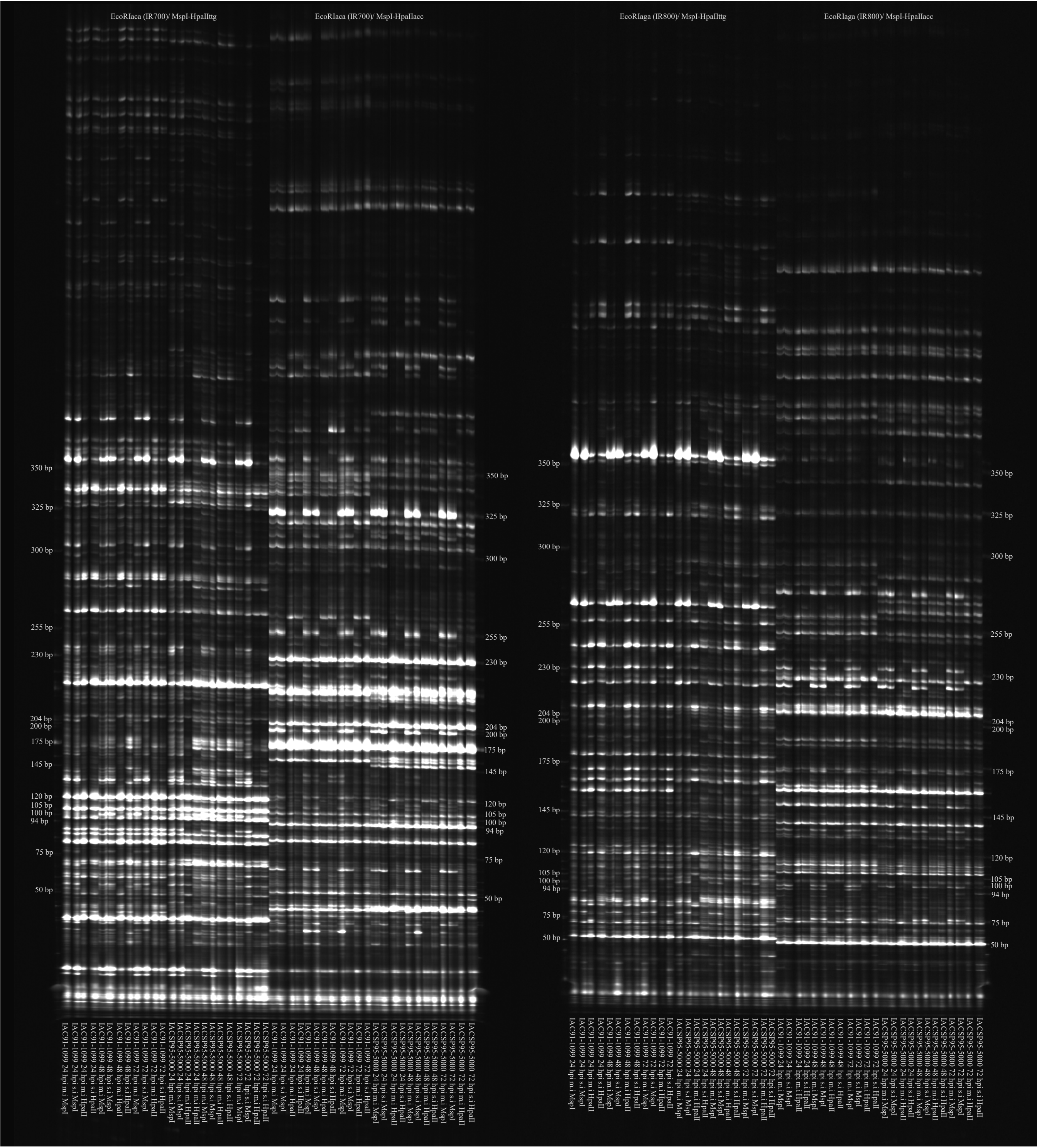

EcoRIaca (IR700)/ MspI-HpaIIttg
EcoRIaca (IR700)/ MspI-HpaIIacc
EcoRIaga (IR800)/ MspI-HpaIIttg
EcoRIaga (IR800)/ MspI-HpaIIacc
350 bp
325 bp
300 bp
255 bp
230 bp
204 bp
200 bp
175 bp
145 bp
120 bp
105 bp
100 bp
94 bp
75 bp
50 bp

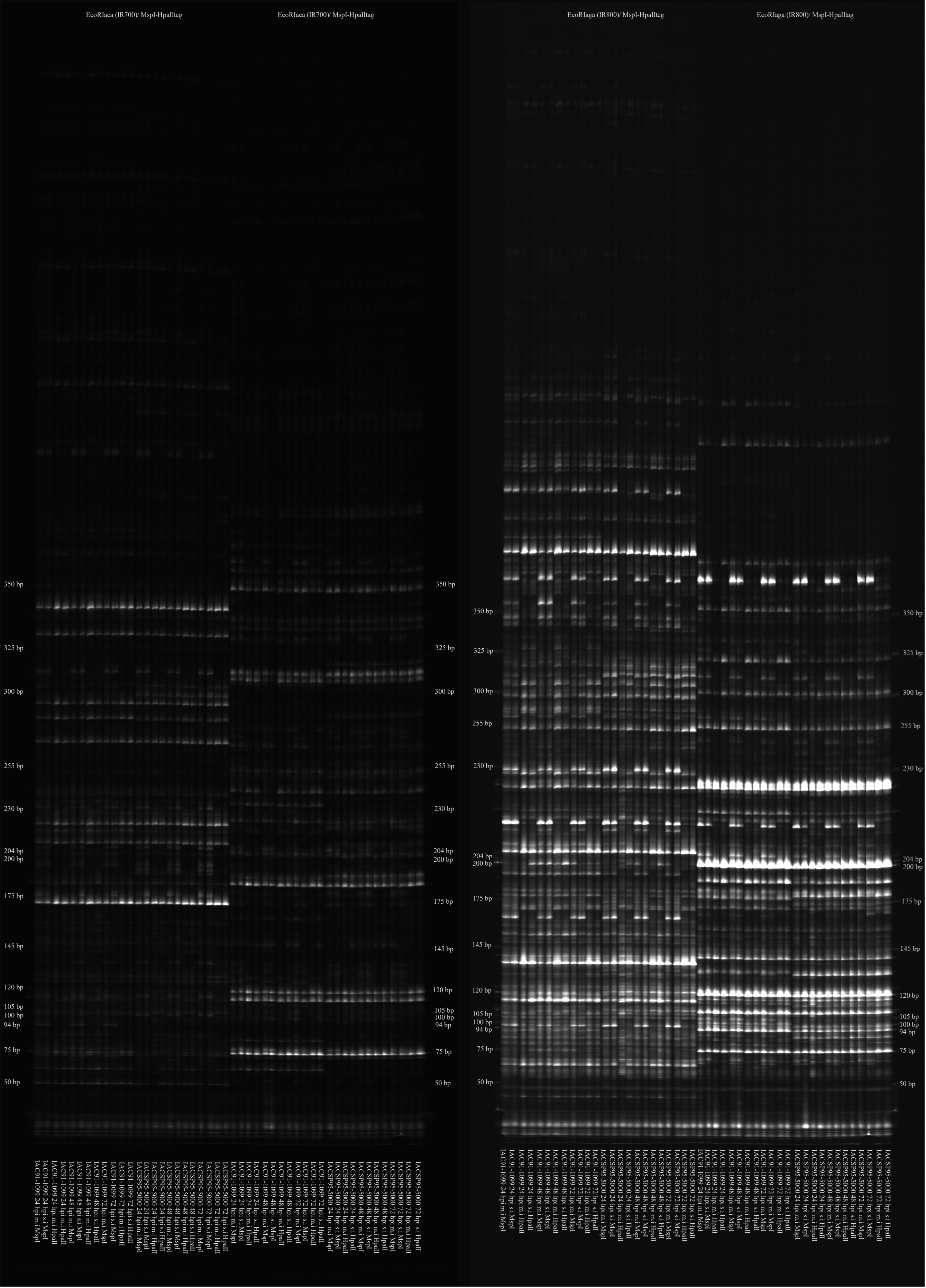
EcoRIaca (IR700)/ MspI-HpaIItcg
EcoRIaca (IR700)/ MspI-HpaIItag
EcoRIaga (IR800)/ MspI-HpaIItcg
EcoRIaga (IR800)/ MspI-HpaIItag
350 bp
325 bp
300 bp
255 bp
230 bp
204 bp
200 bp
175 bp
145 bp
120 bp
105 bp
100 bp
94 bp
75 bp
50 bp
350 bp
325 bp
300 bp
255 bp
230 bp
204 bp
200 bp
175 bp
145 bp
120 bp
105 bp
100 bp
94 bp
75 bp
50 bp
350 bp
325 bp
300 bp
255 bp
230 bp
204 bp
200 bp
175 bp
145 bp
120 bp
105 bp
100 bp
94 bp
75 bp
50 bp
350 bp
325 bp
300 bp
255 bp
230 bp
204 bp
200 bp
175 bp
145 bp
120 bp
105 bp
100 bp
94 bp
75 bp
50 bp

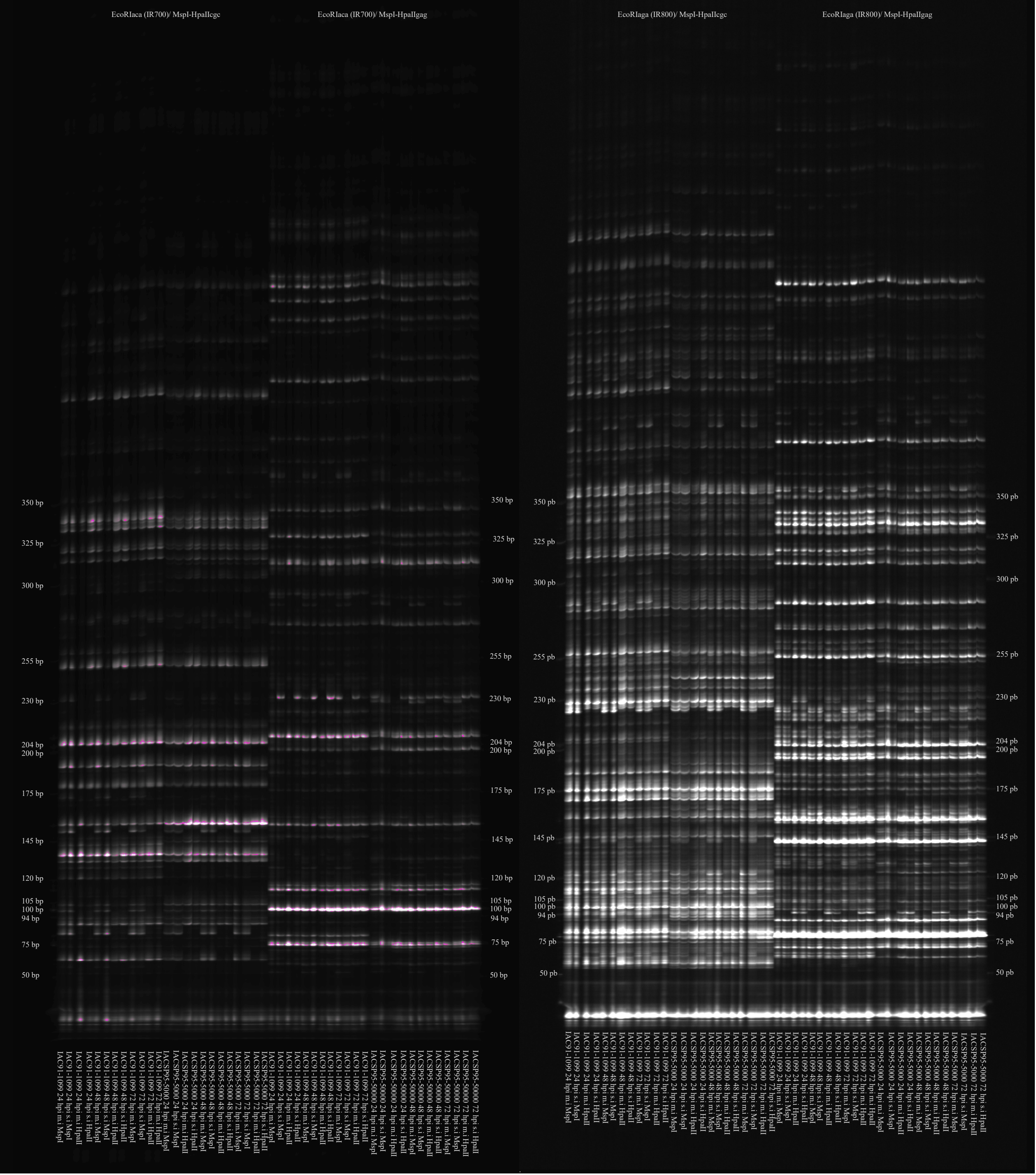
EcoRIaca (IR700)/ MspI-HpaIIcgc
EcoRIaca (IR700)/ MspI-HpaIIgag
EcoRIaga (IR800)/ MspI-HpaIIcgc
EcoRIaga (IR800)/ MspI-HpaIIgag
350 bp
325 bp
300 bp
255 bp
230 bp
204 bp
200 bp
175 bp
145 bp
120 bp
105 bp
100 bp
94 bp
75 bp
50 bp
350 pb
325 pb
300 pb
255 pb
230 pb
204 pb
200 pb
175 pb
145 pb
120 pb
105 pb
100 pb
94 pb
75 pb
50 pb

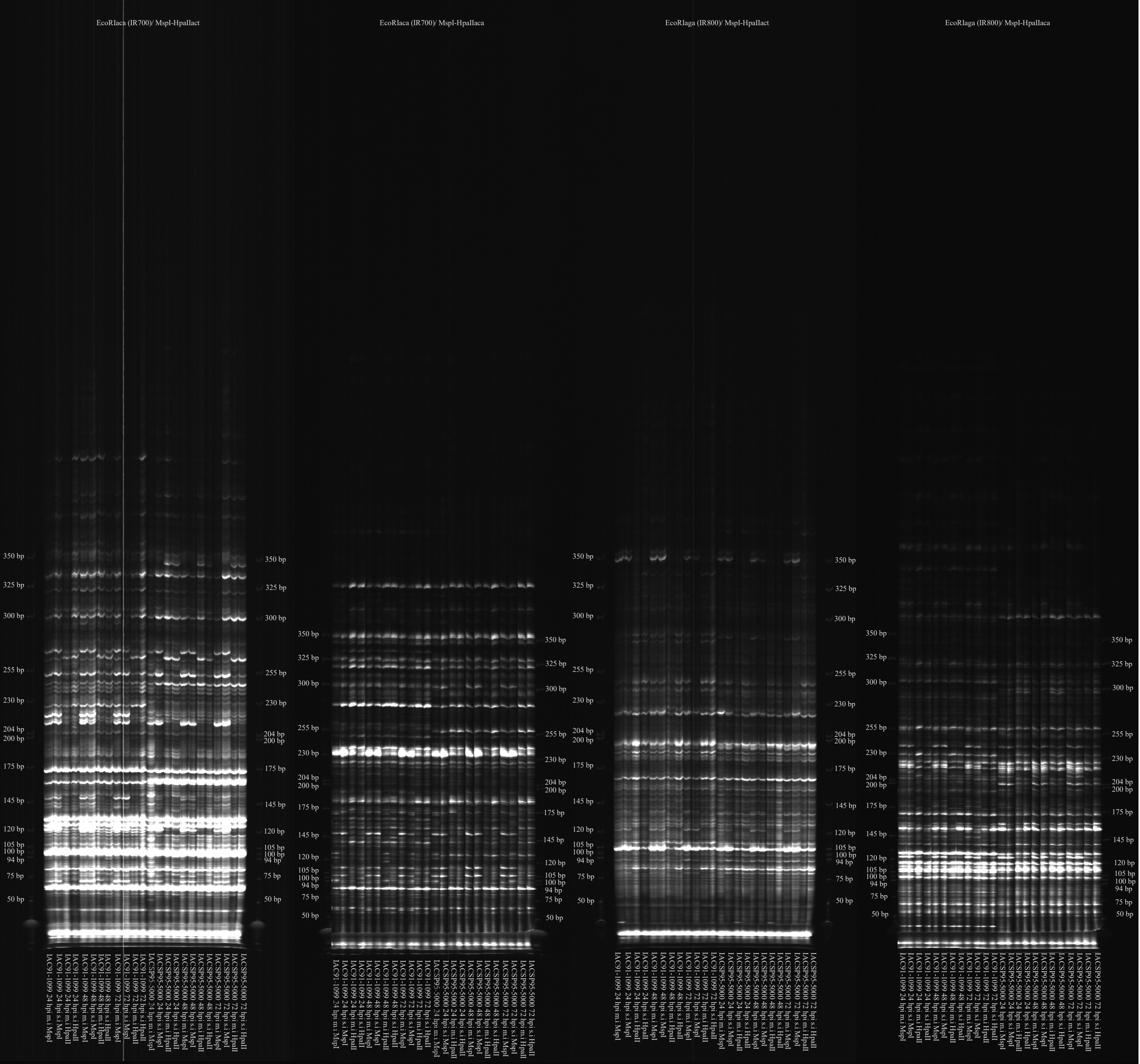
EcoRIaca (IR700)/ MspI-HpaIIact
EcoRIaca (IR700)/ MspI-HpaIIaca
EcoRIaga (IR800)/ MspI-HpaIIact
EcoRIaga (IR800)/ MspI-HpaIIaca

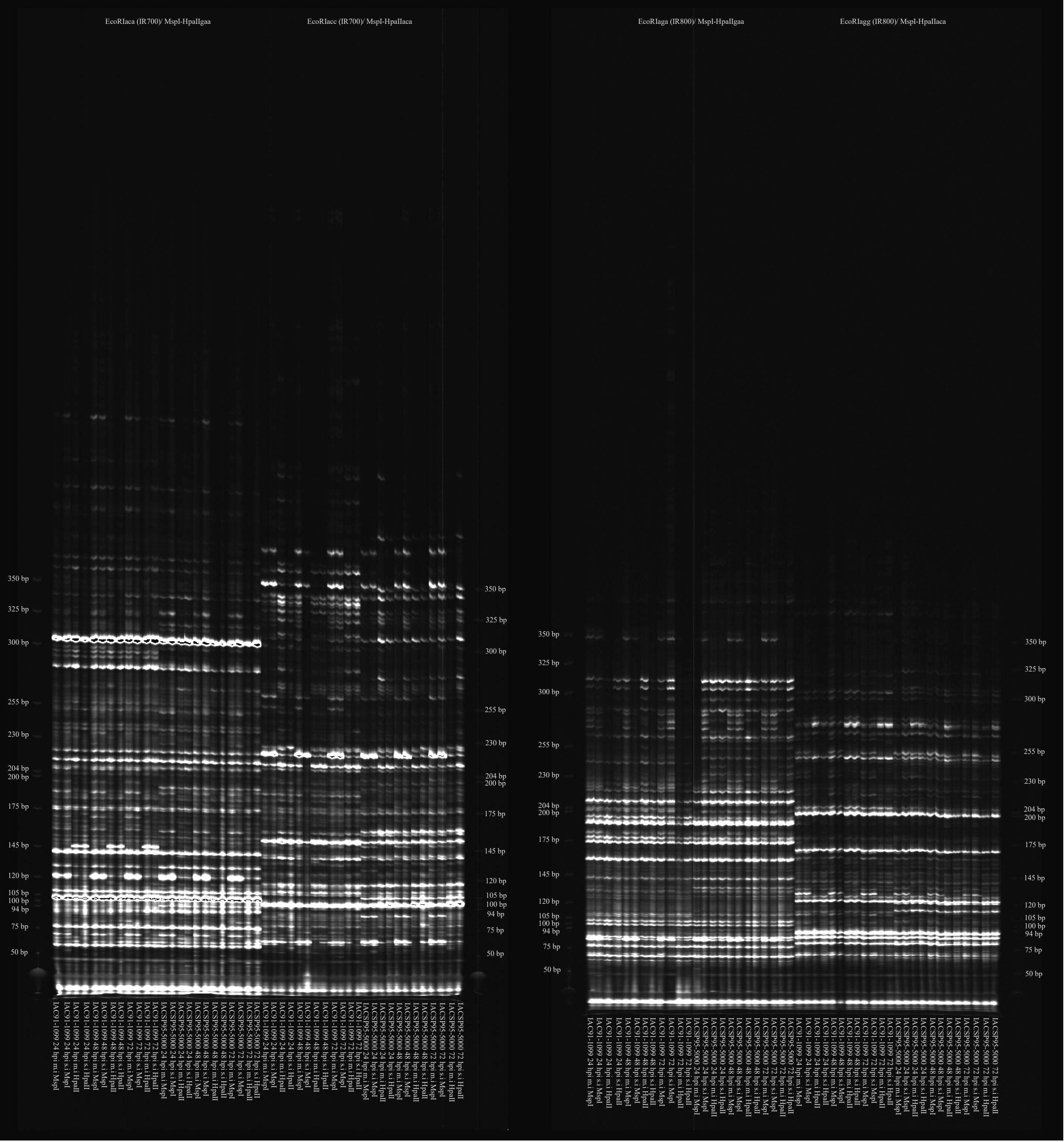

EcoRIaca (IR700)/ MspI-HpaIIgaa
EcoRIacc (IR700)/ MspI-HpaIIaca
EcoRIaga (IR800)/ MspI-HpaIIgaa
EcoRIagg (IR800)/ MspI-HpaIIaca
350 bp
325 bp
300 bp
255 bp
230 bp
204 bp
200 bp
175 bp
145 bp
120 bp
105 bp
100 bp
94 bp
75 bp
50 bp
IAC91-1099 24 hpi m.i MspI
IAC91-1099 24 hpi s.i MspI
IAC91-1099 24 hpi m.i HpaII
IAC91-1099 24 hpi s.i HpaII
IAC91-1099 48 hpi m.i MspI
IAC91-1099 48 hpi s.i MspI
IAC91-1099 48 hpi m.i HpaII
IAC91-1099 48 hpi s.i HpaII
IAC91-1099 72 hpi m.i MspI
IAC91-1099 72 hpi s.i MspI
IAC91-1099 72 hpi m.i HpaII
IAC91-1099 72 hpi s.i HpaII
IACSP95-5000 24 hpi m.i MspI
IACSP95-5000 24 hpi s.i MspI
IACSP95-5000 24 hpi m.i HpaII
IACSP95-5000 24 hpi s.i HpaII
IACSP95-5000 48 hpi m.i MspI
IACSP95-5000 48 hpi s.i MspI
IACSP95-5000 48 hpi m.i HpaII
IACSP95-5000 48 hpi s.i HpaII
IACSP95-5000 72 hpi m.i MspI
IACSP95-5000 72 hpi s.i MspI
IACSP95-5000 72 hpi m.i HpaII
IACSP95-5000 72 hpi s.i HpaII

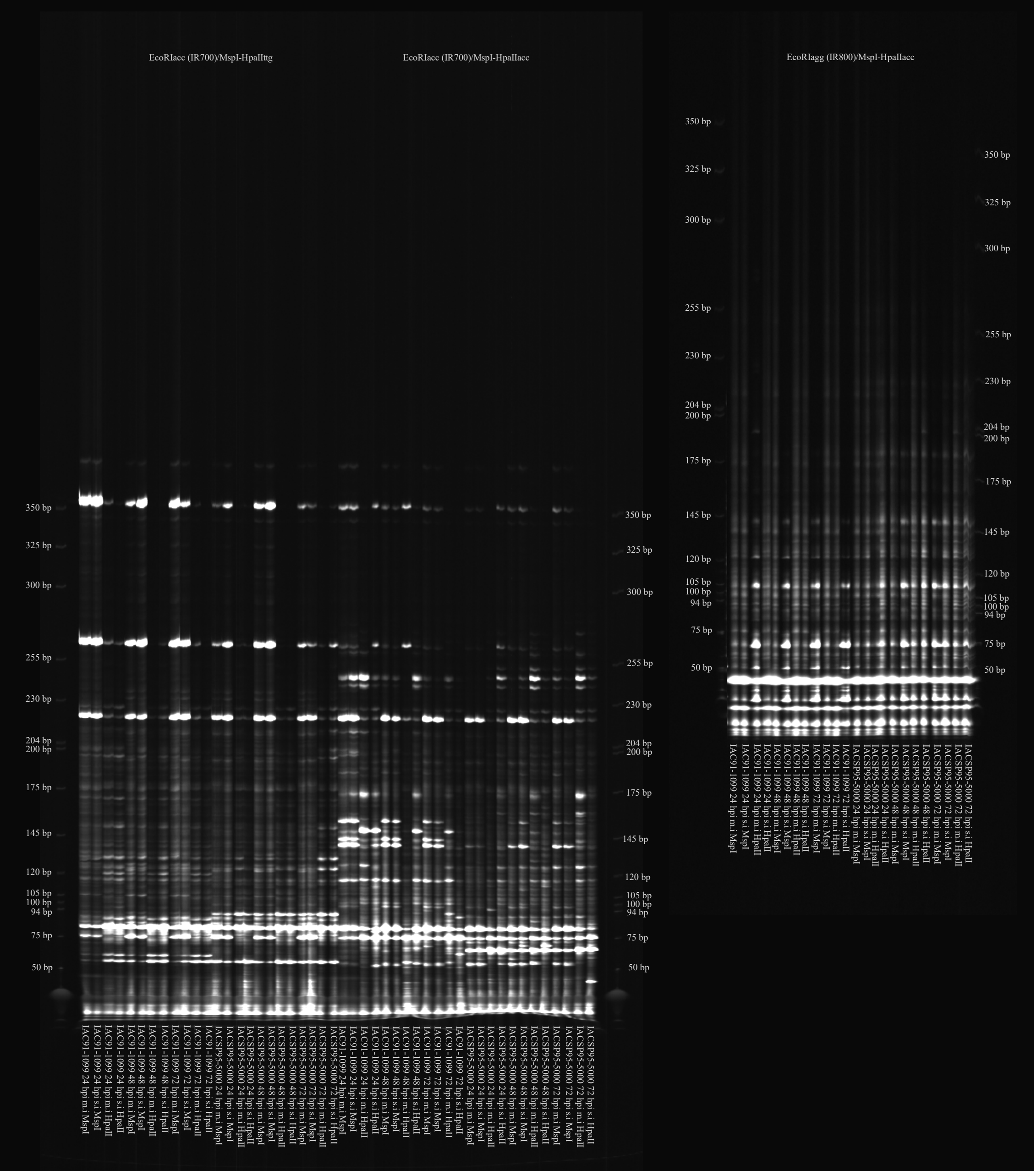
EcoRIacc (IR700)/MspI-HpaIIttg
EcoRIacc (IR700)/MspI-HpaIIacc
EcoRIagg (IR800)/MspI-HpaIIacc
350 bp
325 bp
300 bp
255 bp
230 bp
204 bp
200 bp
175 bp
145 bp
120 bp
105 bp
100 bp
94 bp
75 bp
50 bp
IAC91-1099 24 hpi m.i MspI
IAC91-1099 24 hpi s.i MspI
IAC91-1099 24 hpi m.i HpaII
IAC91-1099 24 hpi s.i HpaII
IAC91-1099 48 hpi m.i MspI
IAC91-1099 48 hpi s.i MspI
IAC91-1099 48 hpi m.i HpaII
IAC91-1099 48 hpi s.i HpaII
IAC91-1099 72 hpi m.i MspI
IAC91-1099 72 hpi s.i MspI
IAC91-1099 72 hpi m.i HpaII
IAC91-1099 72 hpi s.i HpaII
IACSP95-5000 24 hpi m.i MspI
IACSP95-5000 24 hpi s.i MspI
IACSP95-5000 24 hpi m.i HpaII
IACSP95-5000 24 hpi s.i HpaII
IACSP95-5000 48 hpi m.i MspI
IACSP95-5000 48 hpi s.i MspI
IACSP95-5000 48 hpi m.i HpaII
IACSP95-5000 48 hpi s.i HpaII
IACSP95-5000 72 hpi m.i MspI
IACSP95-5000 72 hpi s.i MspI
IACSP95-5000 72 hpi m.i HpaII
IACSP95-5000 72 hpi s.i HpaII

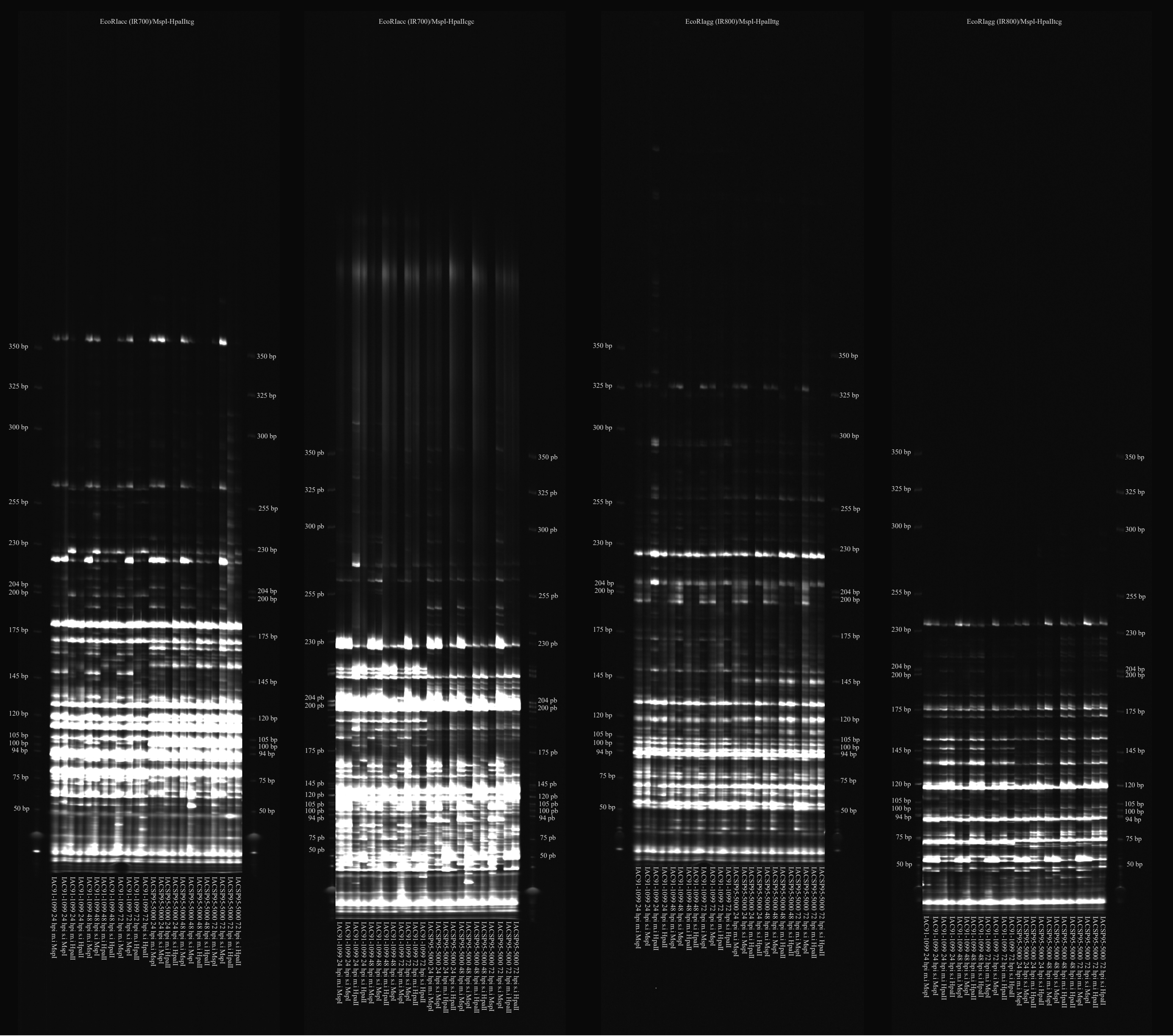
EcoRIacc (IR700)/MspI-HpaIItcg
EcoRIacc (IR700)/MspI-HpaIIcgc
EcoRIagg (IR800)/MspI-HpaIIttg
EcoRIagg (IR800)/MspI-HpaIItcg

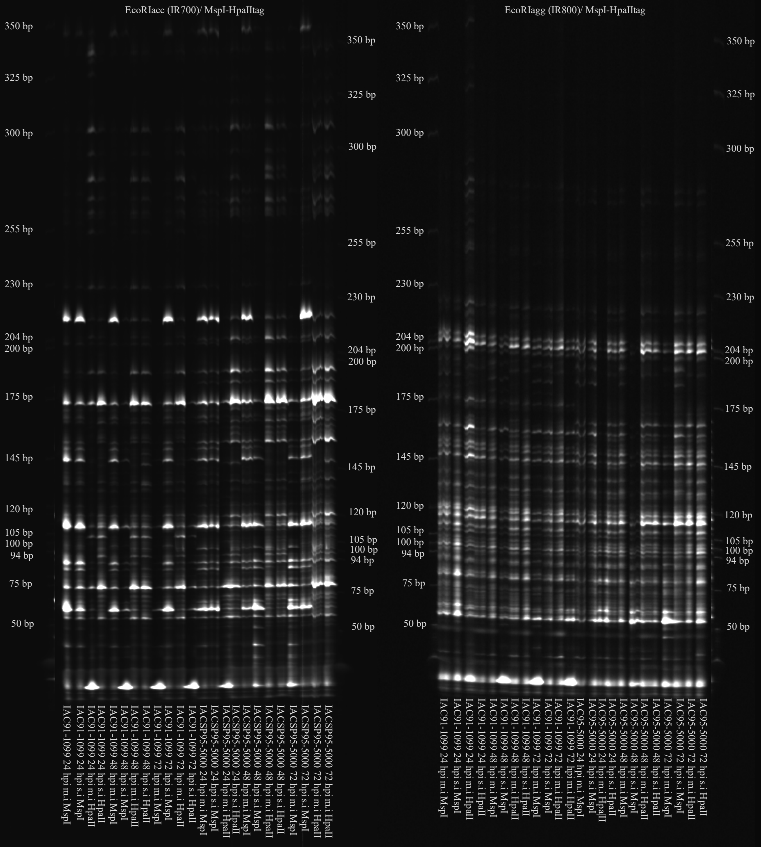
EcoRIacc (IR700)/ MspI-HpaIItag
EcoRIagg (IR800)/ MspI-HpaIItag
350 bp
325 bp
300 bp
255 bp
230 bp
204 bp
200 bp
175 bp
145 bp
120 bp
105 bp
100 bp
94 bp
75 bp
50 bp
IAC91-1099 24 hpi m.i MspI
IAC91-1099 24 hpi s.i MspI
IAC91-1099 24 hpi m.i HpaII
IAC91-1099 24 hpi s.i HpaII
IAC91-1099 48 hpi m.i MspI
IAC91-1099 48 hpi s.i MspI
IAC91-1099 48 hpi m.i HpaII
IAC91-1099 48 hpi s.i HpaII
IAC91-1099 72 hpi m.i MspI
IAC91-1099 72 hpi s.i MspI
IAC91-1099 72 hpi m.i HpaII
IAC91-1099 72 hpi s.i HpaII
IACSP95-5000 24 hpi m.i MspI
IACSP95-5000 24 hpi s.i MspI
IACSP95-5000 24 hpi m.i HpaII
IACSP95-5000 24 hpi s.i HpaII
IACSP95-5000 48 hpi m.i MspI
IACSP95-5000 48 hpi s.i MspI
IACSP95-5000 48 hpi m.i HpaII
IACSP95-5000 48 hpi s.i HpaII
IACSP95-5000 72 hpi m.i MspI
IACSP95-5000 72 hpi s.i MspI
IACSP95-5000 72 hpi m.i HpaII
IACSP95-5000 72 hpi s.i HpaII
IAC95-5000 24 hpi m.i MspI
IAC95-5000 24 hpi s.i MspI
IAC95-5000 24 hpi m.i HpaII
IAC95-5000 24 hpi s.i HpaII
IAC95-5000 48 hpi m.i MspI
IAC95-5000 48 hpi s.i MspI
IAC95-5000 48 hpi m.i HpaII
IAC95-5000 48 hpi s.i HpaII
IAC95-5000 72 hpi m.i MspI
IAC95-5000 72 hpi s.i MspI
IAC95-5000 72 hpi m.i HpaII
IAC95-5000 72 hpi s.i HpaII
